# Cost-effectiveness of prehabilitation for elderly (pre-)frail patients prior to elective surgery compared to standard care - an economic evaluation from a societal perspective

**DOI:** 10.1186/s12916-026-04933-6

**Published:** 2026-05-21

**Authors:** Tanja Rombey, Helene Eckhardt, Wilm Quentin, Zoe Weber, Stefan Buchka, Jörn Kiselev, Verena Loidl, Ulrich Mansmann, Stefan J Schaller, Katrin Schmidt, Claudia Spies, Reinhard Busse, Lukas Schöner

**Affiliations:** 1 Department of Health Care Management, Technische Universität Berlin, Straße des 17. Juni 135, 10623 Berlin, Germany; 2 Chair of Planetary & Public Health, University of Bayreuth, Bayreuth, Germany; 3 Institute for Medical Information Processing, Biometry and Epidemiology (IBE), Faculty of Medicine, Ludwig-Maximilians-Universität München, Munich, Germany; 4 Hochschule Fulda, Department for Health Sciences, University for applied Sciences, Fulda, Germany; 5 Corporate Member of Freie Universität, Department of Anesthesiology and Intensive Care Medicine (CCM/CVK), Charité – Universitätsmedizin Berlin, Humboldt Universität zu Berlin, Berlin, Germany; 6 Department of Anaesthesia, Intensive Care Medicine and Pain Medicine, Clinical Division of General Anaesthesia and Intensive Care Medicine, Medical University of Vienna, Vienna, Austria

**Keywords:** Frailty, Prehabilitation, Surgery, Economic evaluation, Cost-effectiveness, Perioperative medicine

## Abstract

**Background:**

Frailty syndrome is highly prevalent among elderly surgical patients, increasing their risk of experiencing perioperative complications and developing long-term disability. Prehabilitation may reduce these risks by optimising patients’ physiological reserves prior to surgery. The PRAEP-GO trial, a multicentre randomised controlled outcome assessor-blinded trial conducted in Germany, evaluated the (cost-)effectiveness of prehabilitation for older (pre-)frail patients undergoing elective surgery compared to standard care.

**Methods:**

The economic evaluation comprised (i) a cost-utility analysis with the quality-adjusted life year (QALY) as the outcome and (ii) a cost-effectiveness analysis. The latter used the change in level of care dependency from baseline to 12 months postoperatively (deterioration versus no deterioration) as the primary effect measure and the WHO Disability Assessment Schedule (WHODAS 2.0) at 12 months as secondary effect measure. We calculated incremental cost-effectiveness ratios (ICERs) and determined the probability of cost-effectiveness at arbitrary willingness-to-pay thresholds. The main analysis was an intention-to-treat analysis. Additionally, we performed complete case, per-protocol and sensitivity analyses as well as subgroup analyses.

**Results:**

The cost-utility analysis yielded an ICER of 45,547 EUR per QALY gained, indicating a 52% probability of cost-effectiveness at a willingness-to-pay of 50,000 EUR. The cost-effectiveness analysis yielded an ICER of 27,197 EUR per patient with a deterioration in care dependency level prevented, with a 69% probability of being cost-effective at 50,000 EUR. Regarding the QALY, the probabilities were higher in the per-protocol analyses, which included patients who received at least 15 sessions of prehabilitation and those who additionally underwent the planned surgery. The cost-effectiveness analysis based on the WHODAS 2.0 yielded an ICER of 1,241 EUR per point gained. Subgroup analyses revealed that the intervention is cost-effective for patients who received prehabilitation in an outpatient setting compared to a propensity-score matched control.

**Conclusions:**

The intervention was found to be more effective, but also more costly than standard preoperative care. Cost-utility was higher when the intervention was completed as intended. It may be cost-effective in patients who received prehabilitation in an outpatient setting. Future efforts should therefore prioritise optimising adherence and ensuring effective outpatient delivery.

**Trial registration:**

Economic evaluation: OSF Registries (osf.io/ecm74), PRAEP-GO trial: ClinicalTrials.gov (NCT04418271).

**Supplementary Information:**

The online version contains supplementary material available at 10.1186/s12916-026-04933-6.

## Background

In the past 25 years, health expenditure per capita has nearly doubled in high-income countries [1] and is projected to accelerate further [2]. One reason for increasing health expenditures is the demographic change, particularly the ageing population [2]. In Germany, it is estimated that by 2050, 22% of the population will be aged 70 years or older [3]. Due to their higher morbidity, elderly adults are more likely to undergo medical interventions, such as surgery [4–6]. Simultaneously, they face an increased risk of perioperative complications [7], leading to increased morbidity and long-term disability [8], entailing substantial costs to the healthcare and care sectors [9].

Frailty has emerged as a significant factor contributing to negative outcomes in geriatric surgical patients [10–12]. Fried et al. 2001 characterised frailty syndrome by the presence of three or more of the following symptoms: unintentional weight loss, self-reported exhaustion, muscle weakness, slow walking speed, and low physical activity [13]. While cognitive impairment is not part of this definition, it is sometimes considered in broader frailty concepts [14] or described under the term “cognitive frailty” [15]. Despite its well-established role as a predictor of adverse outcomes, frailty is not yet systematically assessed in most health systems and thus insufficiently addressed in standard care [16, 17].

Prehabilitation is a relatively new concept of care, which is defined as “a process from diagnosis to surgery, consisting of one or more preoperative interventions […], that aims to enhance functional capacity and physiological reserve to allow patients to withstand surgical stressors, improve postoperative outcomes, and facilitate recovery” (p. 310) [18]. Outside the surgical context [17], stressors include invasive diagnostic procedures, radiation or chemotherapy [18]. Prehabilitation programmes should typically be multimodal and performed in the days or weeks before the anticipated stressor. The modalities may include physiotherapy, occupational therapy, nutritional counselling, micronutrient supplementation, psychological or neurocognitive support and behavioural risk modification strategies, such as smoking cessation [19].

An overview of 55 systematic reviews has demonstrated that prehabilitation improves postoperative outcomes [18], but the available evidence is of low certainty and study results varied widely depending on context and programme design. A systematic review of the cost-effectiveness of prehabilitation before elective surgery conducted by the authors found some evidence that prehabilitation is cost-effective compared to usual preoperative care [20]. However, the certainty of the evidence was again low due to a relevant risk of publication bias and many studies presenting a high risk of bias and/or low methodological quality. Evidence on prehabilitation particularly for (pre-)frail populations is limited. Existing studies are often characterised by small sample sizes, heterogeneous interventions and methodological limitations, resulting in inconclusive findings [21–24].

To address this evidence gap, the PRAEP-GO trial - a multicentre randomised controlled outcome assessor-blinded trial conducted in Germany - was designed [25, 26]. The trial aimed to evaluate the (cost-)effectiveness of frailty screening, a shared decision-making conference and multimodal prehabilitation for older (pre-)frail patients undergoing elective surgery, compared with standard preoperative care [27].

### Objectives

We conducted a prospectively planned economic evaluation alongside the PRAEP-GO trial to assess the cost-utility and cost-effectiveness of the intervention from a societal perspective. We hypothesised that it would be cost-effective compared to standard care. The ultimate objective of this evaluation was to provide evidence to inform health policy decisions regarding the potential reimbursement of such a prehabilitation programme within the statutory health insurance system in Germany.

## Methods

### Study design

This economic evaluation was carried out alongside the PRAEP-GO trial, a multicentre randomised controlled outcome assessor‑blinded trial performed in 19 trial centres across Germany with a 12-month follow-up [27]. The trial had been registered at ClinicalTrials.gov (NCT04418271) prior to inclusion of the first patient. Ethical approval was granted by the ethics committees of Charité – Universitätsmedizin Berlin (EA1/225/19), and written consent was obtained from each participant prior to their inclusion in the study.

The economic evaluation was planned from the start of the project and did not depend on the clinical trial results given the potential for cost savings at comparable effectiveness. It was conducted according to a published protocol [28], which was pre-registered in OSF registries before closure of the database [29]. Changes from the protocol are documented in Additional file 1: Table S1. Development of the methods was based on the current ISPOR guidelines for cost-effectiveness analysis alongside clinical trials [30], standard works on health economic evaluation [31, 32], and a systematic review of the cost-effectiveness of prehabilitation before elective surgery conducted by the authors beforehand [20]. Reporting of this manuscript followed the CHEERS 2022 checklist [33].

### Study population

Inclusion criteria for the PRAEP-GO trial were all the following:

1. Patient aged 70 years and older
2. Indication for an elective surgery or interventional procedure
3. Expected duration of anaesthesia ≥ 60 min
4. Pre-frail or frail according to criteria by Fried et al. 2001 [13]
5. Enrolled in statutory health insurance

Exclusion criteria were any of the following:

1. Severe cardiac or pulmonary disease.
2. Indication for brain surgery or an intracranial procedure.
3. Moribund patient.
4. Language barrier.
5. Participation in another interventional trial.

Further details on the methods of the trial can be found in the published study protocol [25, 26].

### Setting and location

The 19 trial centres were hospitals that offer care in the statutory healthcare system in the vicinity of one of the three participating university hospitals (which ensured patient follow-up). The university hospitals were located across Germany.

### Comparators

Before randomisation, all patients were screened for frailty to determine their eligibility for the trial. The criteria by Fried et al. 2001 were applied [13], adapted for a European population [10, 17]. Patients were classified as pre-frail if they fulfilled one of the following criteria and frail if they fulfilled two or more: unintentional weight loss, self-reported exhaustion, weakness (based on grip strength), slow walking speed, and low physical activity. Eligible patients were then randomised into the intervention group (IG) or control group (CG).

Both groups received standard perioperative care for their surgery or interventional procedure, as determined and scheduled by their treating physicians. Preoperatively, this typically involved completing imaging and relevant blood tests, a prehospital screening for resistant bacteria, and an anaesthesia work-up. Patients in the CG then underwent their elective surgery. During the operation, standard protocols for each procedure, as implemented locally by each surgical department, were followed for both groups. Following surgery, patients in both groups received the standard postoperative care relating to their procedure. This included decisions on planned intensive care unit stays, analgesia, oral intake, mobilisation, breathing therapy, and physiotherapy, and involved enhanced recovery after surgery (ERAS) protocols where available.

Patients in the IG received the intervention of interest prior to their elective surgery, which consisted of:

1. a shared decision-making process that determined the modalities and setting of.
2. a personalised, three-week multimodal prehabilitation.

The shared decision-making process applied the three-talk model (choice talk, option talk, and decision talk) [34]. The decision talk consisted of a multiprofessional conference involving physicians (anaesthesiologists, surgeons, geriatricians and/or general practitioners), a therapist (physiotherapist, occupational therapist, and/or nutritionist) or a nurse, as well as the patient and a family member if desired. The conference was held following inclusion in the trial and the baseline visit, which included several functional assessments relevant for a geriatric surgical population. The aim of the conference was to define patient-centred prehabilitation goals for prehabilitation and decide on the setting and modalities of prehabilitation.

The multimodal prehabilitation programme included 30 supervised therapy sessions, lasting 30 min each, which were spread across 3 weeks (2 sessions per day, 5 times per week). Additionally, patients were recommended to perform 18 additional unsupervised sessions. The modalities included exercise therapy, nutrition counselling, occupational therapy, speech and language therapy, and cognitive or psychological support. The settings included: mobile prehabilitation (where a therapist visited the patients), outpatient prehabilitation (where the patients visited a therapist’s office), partial inpatient prehabilitation (where the patients received care at a rehabilitation centre during the day), or inpatient prehabilitation (where the patients stayed in the hospital overnight). All prehabilitation centres were instructed by the same trainer and received a prehabilitation manual. The instructions for exercise therapy were based on recommendations for exercise and physical activity for older adults [35, 36], and for nutrition counselling on guidelines on clinical nutrition and hydration in geriatrics [37, 38], and clinical nutrition in surgery [39].

For the purpose of the economic evaluation, frailty screening was considered part of the intervention (only for the IG), because, at the time of the study, it was not standard care in Germany. Nowadays, the European Society of Anaesthesiology and Intensive Care recommends using frailty screening if frailty is suspected, and to have the patient evaluated by a geriatrician when possible [40]. Based on the logic that only a positive screening result can lead to inclusion in the study, patients from the CG also underwent frailty screening, but we did not consider this part of their care, as they were only to receive standard care.

### Perspective

We adopted a societal perspective to evaluate the cost-effectiveness of the intervention because it provides a comprehensive assessment of both direct and indirect costs. This included informal caregiving, which is particularly relevant in a geriatric population. By capturing the wider economic impact on society, this approach ensures that the analysis reflects the full societal burden of the intervention, which is critical for informing policy decisions and optimising resource allocation. In addition, we performed an economic evaluation from the perspective of the statutory health insurance (payer perspective) [41] and from the provider perspective. These will be published separately as they used a different database (claims data from the statutory health insurance), resulting in significant differences in the methods employed.

### Time horizon and discount rate

In line with the PRAEP-GO trial, the time horizon started with the frailty screening and ended after 12 months of follow-up. Extrapolation beyond this time frame was not appropriate due to the lack of long-term data from the trial and uncertainty about the sustained effects of the intervention beyond the initial follow-up period. Due to the short time horizon of just over one year, no discounting was performed.

### Selection, measurement and valuation of clinical effectiveness outcomes

The quality-adjusted life year (QALY) was chosen as the primary effect measure for the cost-utility analyses because it is a composite endpoint consisting of quality of life and length of life, both of which are relevant to patients. Furthermore, it is not disease-specific and internationally recognised, enabling comparisons between different diseases, interventions and healthcare systems. Health-related quality of life was measured using the EQ-5D-5 L questionnaire [42]. It was assessed at 3, 6, 9 and 12 months after surgery. The QALY was calculated using the EQ-5D-5 L results from each time point and valuated using predicted utilities of the German general population [43]. If the patient died, their utility was set to 0 from the date of death (obtained from the study documentation or from population register queries). The worst possible value was a utility of -0.661 for an EQ-5D-5 L health state of 55,555 and the best possible value was a utility of 1 for an EQ-5D-5 L health state of 11,111. The mean value from the four assessment time points was calculated. Accordingly, a QALY of 1 represented one year of full quality of life.

In line with the primary endpoint of the trial, the change in care dependency level from baseline to 12 months postoperatively was chosen as the primary effect measure for the cost-effectiveness analyses. The level of care dependency was assessed using a Germany-specific standardised tool (“Neues Begutachtungsassessment”, NBA) [44] that is used by the statutory health insurance to assess their patients’ need for professional care, which then determines their level of care dependency. The NBA has six domains:

1. mobility,
2. cognitive and communication abilities,
3. behavioural and psychological issues,
4. self-dependence,
5. disease-specific demands and burdens, and.
6. daily routine management and social interactions.

The level of care dependency can range from 0 (no care dependency) to 5 (highest level of care dependency). For the economic evaluation, the change in level of care dependency was dichotomised (deterioration yes/no) and expressed as a proportion. If a patient died throughout the study, their care dependency level at 12-months was set to 5, and thus automatically resulted in a deterioration.

Functional health status at 12 months postoperatively, as measured by the World Health Organisation Disability Assessment Schedule (WHODAS) 2.0 [45], was chosen as a secondary effect measure for cost-effectiveness analysis because it measures similar domains to the NBA (understanding and communication, mobility, self-care, interaction with others, activities of daily living, and participation in social life), but is used globally, allowing comparisons with other healthcare systems. The 12-item version of the questionnaire and ‘simple scoring’ were used, with scores ranging from 12 (best) to 60 (worst). If the patient died, their value was set to 60.

### Measurement and valuation of resources and costs

The total costs consisted of three main blocks:

1. intervention costs,
2. costs of the index hospital stay, i.e. the hospital stay for the elective surgery, and.
3. costs for resource use during the follow-up period of 12 months postoperatively.

All costs were expressed in Euro (EUR) 2019/2020 unless otherwise stated. When a patient died, their costs were set to zero from the date of death. Details on the measurement and valuation of resources and costs, including unit costs, can be found in Additional file 1: Table S2.

Data on the resources used to carry out the intervention were collected from the study documentation, including the number of patients screened for frailty, the number and type of professionals participating in the shared decision-making conference, whether a family member participated in the shared decision-making conference, the setting of prehabilitation, the number of sessions and the type of professionals involved. Training costs for staff and prehabilitation centres were excluded from the intervention costs, as these vary substantially depending on the number of participating hospitals and the number of patients treated at each hospital and prehabilitation centre and are therefore highly dependent on the scale of implementation. Thus, the estimates reflect delivery under established implementation conditions and do not include the full costs of initial implementation. Although patients in the CG underwent frailty screening as a necessary step to determine their eligibility for the trial, the intervention costs in the CG were set to zero as the screening did not generate any additional clinical benefit for these patients and did not represent the standard of care at the time of the study.

Data on the index hospital stay were provided directly by the hospitals (billing data) using a standardised format [46] and supplemented by the study documentation. Co-payments are not included in the medical bills (which are covered by the statutory health insurance). Thus, we added co-payments manually (10 EUR per day, for a maximum of 28 days per calendar year), assuming that 50% of the patients were exempt from these. Investment costs were calculated using a reference value per case calculated by the German National Institute for the Reimbursement of Hospitals [47]. Costs were set to zero for any patients who did not undergo surgery and therefore did not have an index hospitalisation.

Data on resource use during the follow-up were collected as part of the clinical outcome collection at 3, 6, 9 and 12 months postoperatively using a Germany-specific standardised cost questionnaire tailored to elderly patients [48]. The questionnaire comprises 28 items, 16 of which assess resource use. These cover visits to physicians (Q1) and therapists (Q2), use of mobile nursing care (Q3), household assistance (Q4), informal care by family, friends, acquaintances, or neighbours (Q5), days in day care (Q6), and short-term care (Q7) over the previous three months. Q8 asks about benefits from statutory long-term care insurance, Q9 about medication use in the past week. Five questions address days in rehabilitation (Q10), day clinics (Q11), hospital stays (Q12), psychiatric facilities (Q13), and use of auxiliary aids (Q14) over the previous 12 months; these were adapted to refer to the previous three months. Q15 asks about the patient’s current housing situation, and Q16 whether it had changed in the past 12 months due to their health; both questions were also adapted to a three-month recall period. The remaining 12 questions of the questionnaire collect demographic and miscellaneous data.

For valuation, we used the 2020 values of standardised unit costs from a societal perspective developed specifically for use in combination with this questionnaire [49, 50]. These were calculated based on public reports from the statutory health insurance and relevant healthcare provider associations, as well as official national statistics. For instance, the unit cost per visit to a specialist was derived from the quarterly reports of the Federal Association of Statutory Health Insurance Physicians (“Honorarberichte”) [51], which provides the total sum of claims and the total number of visits per quarter. In the absence of consensus on how to appropriately measure and value informal care [52], we valued it using the substitution cost approach [49], applying the standardised unit costs for wages that would have been incurred had professional caregivers provided the care instead.

### Outcome measures

To calculate the incremental costs and effects of the intervention, we calculated the mean difference (MD) in costs and the MD or the percentage difference (%-diff, i.e., the absolute risk reduction) in clinical effectiveness outcomes between the IG and CG. The values for the probability of deterioration of care dependency level (DCDL) and the WHODAS 2.0 score were inverted, because here lower values were preferable.

The primary endpoint of the economic evaluation was the relationship between incremental costs and effects, which was used to determine cost-effectiveness. The intervention dominated the control when it was more effective and less costly, and was dominated by the control when it was less effective and more costly. For unclear situations, e.g., when the intervention was more effective but also more costly, we calculated the incremental cost-effectiveness ratio (ICER) using the incremental costs as the numerator and incremental effects as the denominator. Thus, the ICER reflects the additional costs incurred for an additional unit of benefit, such as one QALY.

Costs were presented using means with standard deviation (SD) and medians with interquartile ranges (IQR) between the first and third quartiles. Clinical effectiveness outcomes were presented as means with SD or absolute numbers (n) and proportions (in percent).

### Analytics and assumptions

All analyses were performed using R software [53] using different R packages, such as “dyplr” [54], “tidyr” [54] and, for creating figures, “ggplot2” [55]. Code writing was supported by ChatGPT (Models: GPT-3.5, GPT-4o and GPT-4o mini, OpenAI, San Francisco).

The sample size of the PRAEP-GO trial was calculated to have a power of 80% to detect the expected change in care dependency level (primary endpoint), detailed in the trial protocol [25], when analysed with a Wilcoxon-Mann-Whitney rank sum test with a 0.05 two-sided significance level [56]. This resulted in 470 patients per group. It was additionally estimated that 2.5% of patients would receive an incorrect treatment allocation, and 30% would be lost to follow-up after 12 months. Thus, the targeted sample size for the trial was 1,378 patients [27].

As specified in the protocol [28], we anticipated that the trial’s broad inclusion criteria might lead to differences in patient characteristics between the intervention and CGs, despite randomisation. Therefore, we calculated the standardised mean difference (SMD) for relevant demographic variables (age, sex, Charlson Comorbidity Index [57], index department (as proxy for type of surgery), surgical risk [58]) to determine the need for adjustment for any of these variables (cut-off was an SMD of ≥ 10%). However, this revealed no need for adjustment (see Table 1 and Additional file 1: Table S3).

To reduce bias resulting from the influence of extreme costs on mean values, costs were winsorised from the 97.5th percentile for each cost block. We chose this cut-off arbitrarily because it would only correct for extreme outliers that would have significantly influenced the results, while only having to manipulate a small proportion of the data. The intervention costs were not winsorised, as no outliers were expected nor observed. To assess structural uncertainty, a sensitivity analysis (SA) without winsorising the costs was performed to assess its impact on the results.

We performed a complete case analysis excluding all patients for whom cost or effectiveness data were missing at any time point. For all other analyses, missing cost and effectiveness data were imputed, assuming they were missing at random. Imputation was performed for all cost blocks and outcome variables across all study visits relevant to the economic evaluation. Imputed values were combined across datasets at the individual level prior to analysis, i.e. total costs and aggregated outcomes were subsequently derived from the imputed component-level data. The multiple imputation by chained equations approach was applied using the R package “mice” [59].

Propensity score matching was used for the two per per-protocol analyses and the subgroup analysis by setting to select patients from the CG that matched the remaining patients in the IG using the R package “matchit” [60]. This was necessary, as the CG did not receive the intervention and thus no information on the number of sessions or the setting was available for them. Matching was performed using a 1:1 nearest neighbour approach, based on the following characteristics: age, sex, body mass index (BMI), Charlson Comorbidity Index, frailty status, care dependency level at baseline (not included when analysing DCDL), EQ-5D-5 L utility at baseline, cognition at baseline, WHODAS 2.0 at baseline and recruiting specialty. As these analyses were not performed on the originally randomised population, but rather on populations selected based on post-randomisation variables, they should be interpreted as non-randomised, exploratory analyses, which may be subject to residual confounding.

### Main analyses

The economic evaluation consisted of two types of analyses using different clinical outcomes:

1. Cost-utility analysis using the QALY.
2. Cost-effectiveness analysis using the.
  - Change in level of care dependency as the primary effect measure.
  - WHODAS 2.0 as a secondary effect measure.

For both analysis types, five different analyses were performed:

1. An intention-to-treat analysis using imputed data (ITT).
2. An intention-to-treat analysis using complete data only (CC).
3. A per-protocol analysis of patients who had performed at least 15 supervised prehabilitation sessions using imputed data and propensity score matching (see below) to match patients from the CG (PP15).
4. A per-protocol analysis of PP15 patients who additionally had undergone surgery using imputed data and propensity score matching to match patients from the CG (PP15OP).
5. A sensitivity analysis using costs without winsorisation (SA-Costs).

For the cost-effectiveness analysis, an additional sensitivity analysis was performed, in which patients who had died were excluded, in order to assess the impact of the otherwise applied ‘worst-score-at-death’ assumption that would otherwise have been applied (SA-Effects).

### Subgroup analyses

The following pre-specified subgroup analyses were performed:

1. Age (younger patients with an age < median vs. older patients with an age ≥ median).
2. Sex (male vs. female).
3. Type of surgery (orthopaedic surgery including neurosurgery of the spine vs. heart surgery or cardiac procedure vs. tumour surgery vs. other surgeries).
4. Frailty status (pre-frail vs. frail).
5. Setting of prehabilitation (outpatient setting including mobile and part-inpatient prehabilitation vs. inpatient setting) using propensity score matching (see below) to match patients from the CG.

Additionally, the following exploratory post-hoc subgroup analyses were performed to investigate the role of patients’ care dependency level and cognitive function at baseline, both of which turned out to be lower than we expected:

1. Care dependency level at baseline (0 vs. ≥ 1).
2. Cognition at baseline (normal (Montreal Cognitive Assessment (MOCA) score ≥ 26) vs. abnormal (MOCA score < 26) [61]).

For the WHODAS 2.0, only the ITT analysis using imputed data and no subgroup analyses were performed, as it served as a secondary effect measure for the cost-effectiveness analysis.

### Characterising uncertainty

To assess uncertainty around the cost and effect estimates and consider possible deviations from distribution assumptions, we performed bootstrapping with 10,000 resamples to calculate 95% confidence intervals (CIs) for the mean differences using the R package “boot” [62]. The same resamples were used to construct incremental cost-effectiveness scatter plots of the two primary effect measures, visualising the distribution of simulated outcomes around the point estimates, as well as cost-effectiveness acceptability curves, which indicate the probability of the intervention being cost-effective at different willingness-to-pay thresholds. These ranged from 0 to 100,000 EUR and we chose 50,000 EUR as an arbitrary reference point.

### Approach to engagement with patients and others affected by the study

While patients and members of the public were not included in the design of the economic evaluation, clinicians such as anaesthetists and physiotherapists were closely involved. In addition, BARMER, a statutory health insurance provider, contributed to the design of the economic evaluation from the perspective of statutory health insurance. Moreover, we conducted qualitative studies with patients [63] and healthcare professionals [64] from the PRAEP-GO trial to explore barriers and facilitators to implementing the intervention from their perspectives.

## Results

### Study parameters

The clinical results of the PRAEP-GO trial are published elsewhere [27]. Enrolment took place between June 27, 2020, and July 14, 2023, resulting in a total of 1,382 included and randomised patients (692 in the IG vs. 690 in the CG). Of these, 167 patients withdrew consent, 13 were lost to follow-up, and 3 were false inclusions, leaving 1,199 patients in the trial (616 vs. 583). The follow-up was completed on August 1, 2024.

The patients’ baseline characteristics are displayed in Table 1. Just over half of the patients were female, with a median age of 78 years and a median BMI of 27 in both groups. The proportions of pre-frail (63%) and frail (37%) patients were identical, as was the median Charlson Comorbidity Index (= 5). The median MOCA score was 24 in both groups, and 82% vs. 86% of patients had a suspected mild or severe neurocognitive disorder. The majority of patients had a care dependency level at baseline of 0 (76% in the IG and 72% in the CG). The median EQ-5D-5 L utility was 0.7 [0.4, 0.9] in both groups, and the median WHODAS 2.0 score was 24 [18, 30] in the IG and 23 [18, 31] in the CG.

**Table 1:** Baseline characteristics of the study population

Characteristic	Intervention group (*n* = 616)	Control group (*n* = 583)
Age	78 [74, 82]	78 [73, 82]
Female sex	328 (53.2)	325 (55.7)
Body mass index	27.0 [24.0, 30.5]	26.8 [23.8, 30.4]
Frailty status		
Pre-frail	388 (63.0)	369 (63.3)
Frail	228 (37.1)	214 (36.8)
Charlson Comorbidity Index	5 [4, 6]	5 [4, 6]
MOCA score	24 [21, 26]	24 [21, 26]
Pathological MOCA score (< 26)	409 (66.4)	387 (66.4)
Care dependency level		
0	469 (76.1)	422 (72.4)
1	84 (13.6)	100 (17.2)
2	44 (7.1)	37 (6.3)
3	19 (3.1)	20 (3.4)
4	0 (0.0)	3 (0.5)
5	0 (0.0)	1 (0.2)
EQ-5D-5L utility	0.7 [0.4, 0.9]	0.7 [0.4, 0.9]
WHODAS 2.0	24 [18, 30]	23 [18, 31]

Values are presented as median [interquartile range] or n (%)

Abbreviations: MOCA, Montreal Cognitive Assessment; WHODAS, World Health Organization Disability Assessment Schedule

In the IG, 556 (90%) patients received an SDM conference and 519 (84%) had at least one session of prehabilitation. Of those, most patients received prehabilitation in a form of outpatient setting (343 with transportation, 69 without transportation, 23 part-inpatient, 4 mobile; total *n* = 439; 85%) and 80 patients (15%) in an inpatient setting. In 99% of patients, prehabilitation involved exercise therapy, 39% received nutritional counselling, 5% received cognitive and psychological support, and 49% received one or more of the other modalities. The median number of supervised sessions was 29.5 (IQR 26 to 30). Of the 583 patients in the CG, 582 (99.8%) patients received standard care, while one patient (0.2%) received the intervention.

The majority of patients underwent the originally planned index surgery (90% in the IG vs. 88% in the CG). Most surgeries were performed in orthopaedic and trauma departments (53% vs. 50%), followed by ophthalmology (7% vs. 10%) and cardiology (7% vs. 10%).

### Total costs

The total costs included the main cost blocks of the intervention costs, the costs for the index hospital stay and the costs due to resource use in the 12-month follow-up period. An overview of these costs following winsorisation and imputation is displayed in Table 2. An overview of these costs following winsorisation, but without imputation, and following imputation, but without winsorisation can be found in Additional file 1: Table S4 and S5.

**Table 2:** Cost overview following winsorisation and imputation (in EUR)

Cost block	Intervention group (*n* = 616)					Control group (*n* = 538)				
	Mean	SD	Median	Q1	Q3	Mean	SD	Median	Q1	Q3
**Intervention**	**2,414**	**2,837**	**1,546**	**1,025**	**2,122**	Not applicable (**0 EUR**)				
Frailty screening	26	0	26	26	26					
SDM conference*	309	116	332	287	387					
Prehabilitation*	2,079	2,808	1,195	644	1,749					
Mobile (*n* = 4)	831	155	798	747	882					
Outpatient (*n* = 69)	608	222	644	579	713					
Outpatient with transportation (*n* = 343)	1,360	577	1,277	1,006	1,580					
Part-inpatient (*n* = 23)	1,900	399	2,029	1,662	2,254					
Inpatient (*n* = 80)	9,069	924	9,056	8,883	9,217					
Index hospital stay**	**10**,**540**	**8**,**065**	**7**,**996**	**5**,**621**	**11**,**334**	**10**,**852**	**8**,**621**	**7**,**982**	**5**,**105**	**11**,**958**
**12-month follow-up**	**12**,**994**	**18**,**452**	**5**,**914**	**2**,**868**	**14**,**235**	**13**,**849**	**18**,**461**	**6**,**742**	**3**,**461**	**14**,**973**
Hospital (inpatient)	5,580	10,961	607	0	5,742	5,875	11,499	1,012	0	6,121
Hospital (outpatient)	147	423	0	0	0	138	437	0	0	0
Psychiatry	0	0	0	0	0	0	0	0	0	0
Visits to physicians	679	430	616	385	876	689	453	626	355	907
Visits to therapists	708	672	508	191	1,030	715	718	489	190	1,023
Mobile nursing care	677	1,859	0	0	418	886	2,200	0	0	475
Informal care	2,722	11,138	0	0	0	2,879	12,032	0	0	0
Household assistance	106	301	0	0	0	103	289	0	0	0
Help from neighbours etc.	314	1,398	0	0	0	435	1,615	0	0	0
Day care	0	0	0	0	0	0	0	0	0	0
Short-term care	0	0	0	0	0	0	0	0	0	0
Nursing home	228	1,213	0	0	0	137	949	0	0	0
Rehabilitation	1,609	2,022	684	0	3,248	1,754	2,040	1,072	0	3,040
Auxiliary aids	225	423	61	0	184	238	466	61	0	154
**Total costs**	**26**,**621**	**25**,**088**	**18**,**360**	**12**,**312**	**32**,**846**	**25**,**418**	**24**,**617**	**16**,**724**	**11**,**316**	**30**,**169**

*This includes patients who did not receive an SDM conference or prehabilitation, for which the cost equalled 0 EUR

**This includes patients who did not undergo surgery and thus had no index hospital stay, for which the cost equalled 0 EUR

Abbreviations: SD, standard deviation; SDM, shared decision-making; Q1, first quartile; Q3 third quartile

The mean intervention costs were 2,414 ± 2,837 EUR for the IG and 0 ± 0 EUR for the CG, who received standard care (no costs applicable). Prehabilitation was the main cost driver, accounting for 86% of the mean and 77% of the median intervention costs, mainly due to the high costs of inpatient prehabilitation. The mean costs for the index hospital stay were comparable between the groups with 10,540 ± 8,065 EUR in the IG and 10,852 ± 8,621 EUR in the CG. Follow-up costs were lower in the IG (mean 12,944 ± 18,452 EUR) than in the CG (mean 13,849 ± 18,461 EUR), the main cost drivers being hospital stays, informal care and rehabilitation. The mean total costs were 26,621 ± 25,088 EUR and 25,418 ± 24,617 EUR. As is often the case with cost data, which are typically skewed, the median values were generally lower for each cost block. Without imputation the mean total costs were Total costs 19,908 ± 20,063 EUR and 17,730 ± 20,287 (+ 2,178 EUR; Table S4) and without winsorisation the mean total costs were 28,996 ± 31,326 EUR and 29,008 ± 35,463 EUR (-12 EUR; Table S5).

### Cost-utility based on quality-adjusted life year (QALY)

The results of the cost-utility analysis based on the QALY are displayed in Table 3. Mean EQ-5D-5 L utility values at baseline and per follow-up visit are reported in Additional file 1: Table S6. The main analysis, the ITT analysis using winsorised and imputed data, found incremental costs of 1,203 EUR (bootstrapped 95% CI: -1,620; 3,997) and incremental effects of 0.03 QALYs (bootstrapped 95% CI: -0.01; 0.06), resulting in an ICER of 45,547 EUR per QALY gained. The complete case analysis including 223 patients with complete outcome data revealed higher incremental costs than the ITT and found no incremental effects, resulting in a very large ICER of almost 500,000 EUR. The effectiveness of the intervention group was highest in this analysis, but this did not result in higher incremental effects, as it also applied to the control group, which was equally effective here. The non-randomised, exploratory per-protocol analyses revealed lower incremental costs and higher incremental effects compared to the ITT analysis, resulting in smaller ICERs. The sensitivity analysis found a minimal cost reduction with identical incremental effects as the ITT analysis, leading to a situation where the intervention dominates the control by being less costly and more effective.

**Fig. 1:**
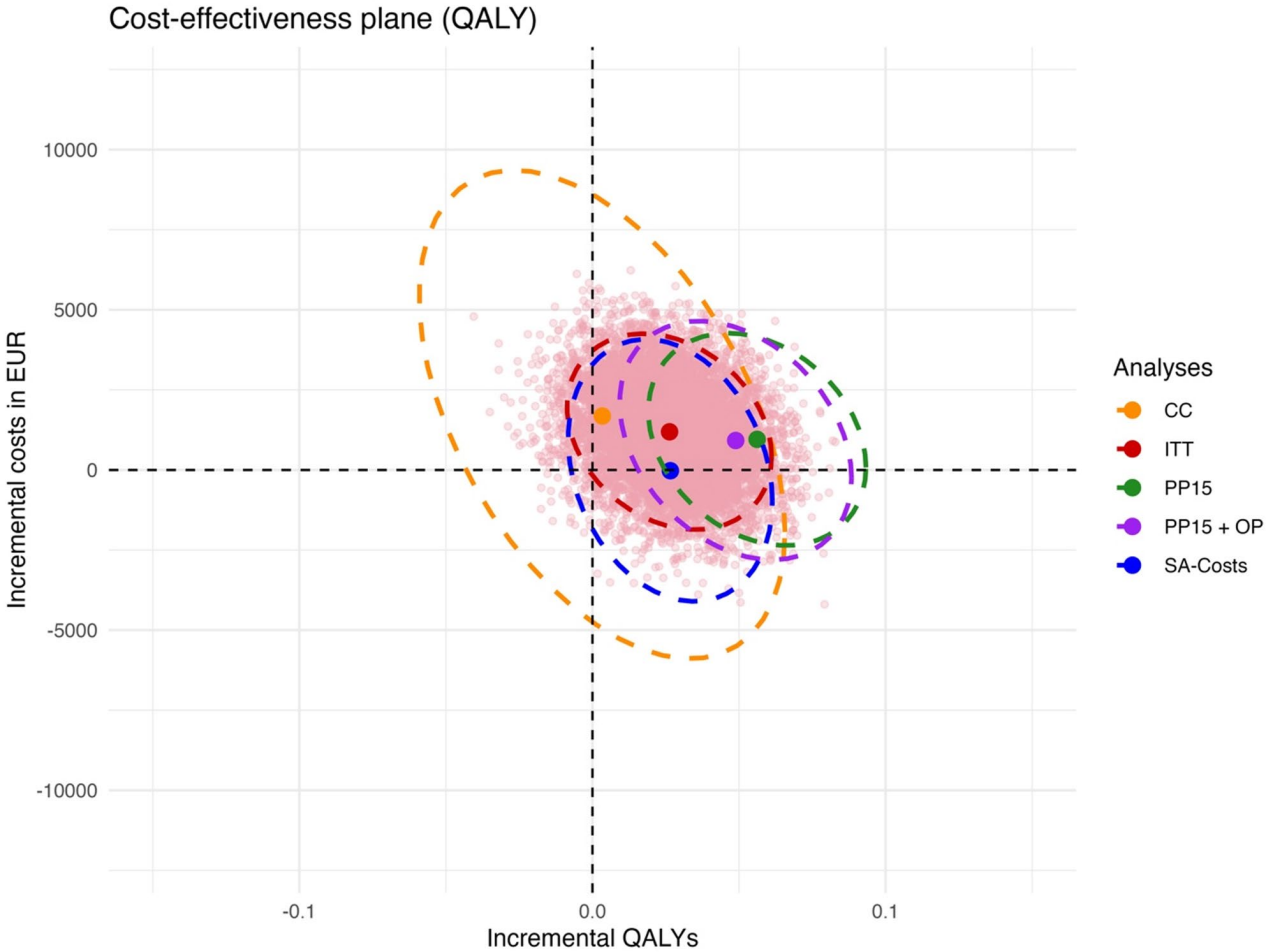
Cost-effectiveness plane showing a scatter plot of the ITT analysis and 95%-CI ellipses of all cost-utility analyses based on the QALY

Figure 1 shows a cost-effectiveness plane with a scatter plot of the ITT analysis in pink and the point estimate and 95%-CI ellipse in red, as well as 95%-CI ellipses of all cost-utility analyses based on the QALY. The majority of points are located in the upper right quadrant, indicating that the intervention is more effective, but more costly. About one-third of the points are located in the lower right quadrant, indicating that the intervention dominates the control by being more effective and less costly. The 95%-CI ellipse for the complete case analysis is the largest due to the smaller number of included patients, indicating the highest level of uncertainty. All ellipses crossed the horizontal axis for the costs, indicating that the intervention might be more costly or less costly. The ITT, CC and SA ellipses also crossed the vertical axis for the effects, but the non-randomised, exploratory per-protocol analyses’ ellipses did not, indicating that the intervention was significantly more effective when only including participants and matched controls who adhered to the intervention.

**Table 3:** Results of the cost-utility analysis based on the quality-adjusted life year (QALY)

Analysis	*N*		Costs IG		Costs CG		Incremental costs		Effects IG		Effects CG		Incremental effects		ICER*
	IG	CG	Mean	SD	Mean	SD	MD	95% CI**	Mean	SD	Mean	SD	MD	95% CI**	EUR/QALY
ITT	616	583	26,621	25,088	25,418	24,617	1,203	-1,620; 3,997	0.72	0.28	0.69	0.29	0.03	-0.01; 0.06	45,547
CC	131	92	28,609	24,684	26,929	27,583	1.679	-5,518; 8;562	0.80	0.23	0.80	0.20	0.00	-0.05; 0.06	497.069
PP15***	473	473	25,879	23,177	24,939	24,714	940	-2,116; 3,976	0.76	0.25	0.70	0.29	0.06	0.02; 0.09	16,752
PP15OP***	391	391	26,596	23,343	25,642	25,120	954	-2,406; 4,315	0.77	0.24	0.72	0.28	0.05	0.01; 0.09	19,520
SA-Costs	616	583	28,996	31,326	29,008	35,463	-12	-3,793; 3,813	0.72	0.28	0.69	0.29	0.03	-0.01; 0.06	Intervention dominates

*ICERs were calculated using the raw values, i.e. without rounding

**Bootstrapped 95% confidence interval using 10,000 resamples

***Propensity score matched, i.e. non-randomised, population

Abbreviations: CC, complete cases; CG, control group; CI, confidence interval; IG, intervention group; ICER, incremental cost-effectiveness ratio; ITT, intention-to-treat; MD, mean difference; OP, operation; PP, per protocol; SA, sensitivity analysis; SD, standard deviation; QALY, quality-adjusted life year

### Cost-effectiveness based on deterioration in care dependency level (DCDL)

The results of the cost-effectiveness analysis based on the DCDL are displayed in Table 4. The main analysis, the ITT analysis using winsorised and imputed data, found incremental costs of 1,203 EUR (bootstrapped 95% CI: -1,596; 4,051) and incremental effects of 4.42% (bootstrapped 95% CI: -0.05; 8.94) patients with prevented DCDL, resulting in an ICER of 27,197 EUR per patient with DCDL prevented. The complete case analysis including 346 patients with complete outcome data found a cost reduction and higher incremental effectiveness than the ITT, resulting in a situation where the intervention dominates the control by being less costly and more effective. The non-randomised, exploratory per-protocol analyses revealed higher incremental costs and higher incremental effects compared to the ITT analysis, resulting in higher, i.e. less favourable, ICERs. The PP15 per-protocol analysis stood out, as it found much higher incremental effects than the ITT analysis, but also more than twice as high the incremental costs. The sensitivity analysis using non-winsorised costs found a minimal cost reduction with identical incremental effectiveness as the ITT analysis, also leading to a situation where the intervention dominates the control. The sensitivity analysis excluding patients who died found similar incremental costs as the ITT analysis but lower incremental effects, resulting in an ICER of 37,689 EUR. The effectiveness of both groups was markedly better when excluding patients with any missing data or patients who died, i.e. in the complete case and SA-Effects analyses.

**Table 4:** Results of the cost-effectiveness analysis based on the deterioration in care dependency level (DCDL)

Analysis	*N*		Costs IG		Costs CG		Incremental costs		Effects IG		Effects CG		Incremental effects		ICER*
	IG	CG	Mean	SD	Mean	SD	MD	95% CI**	*N* with DCDL	%	*N* with DCDL	%	%-diff	95% CI**	EUR/ prevented DCDL
ITT	616	583	26,621	25,088	25,418	24,617	1,203	-1,596; 4,051	108	17.53	128	21.96	4.42	-0.05; 8.94	27,197
CC	197	149	26,802	22,617	27,295	27,143	-493	-6.110; 4,733	15	7.61	22	14.77	7.15	0.54; 14.10	Intervention dominates
PP15***	470	470	26,896	24,268	24,245	22,190	2,651	-282; 5,676	67	14.26	99	21.06	6.81	1.83; 11.58	38,938
PP15OP***	382	382	26,513	23,089	24,985	22,604	1,528	-1,726; 4,795	54	14.14	72	18.85	4.71	-0.56; 9.96	32,427
SA-Costs	616	583	28,996	31,326	29,008	35,463	-12	-3,784; 3,854	108	17.53	128	21.96	4.42	0.02; 9.03	Intervention dominates
SA-Effects	573	534	26,560	25,116	25,260	24,258	1,300	-1,592; 4,169	65	11.34	79	14.79	3.45	-0.53; 7.47	37,689

*ICERs were calculated using the raw values, i.e. without rounding

**Bootstrapped 95% confidence interval using 10,000 resamples

***Propensity score matched, i.e. non-randomised, population

Abbreviations: CC, complete cases; CG, control group; CI, confidence interval; DCDL, deterioration in care dependency level; IG, intervention group; ICER, incremental cost-effectiveness ratio; ITT, intention-to-treat; MD, mean difference; OP, operation; PP, per protocol; SA, sensitivity analysis; SD, standard deviation

**Fig. 2:**
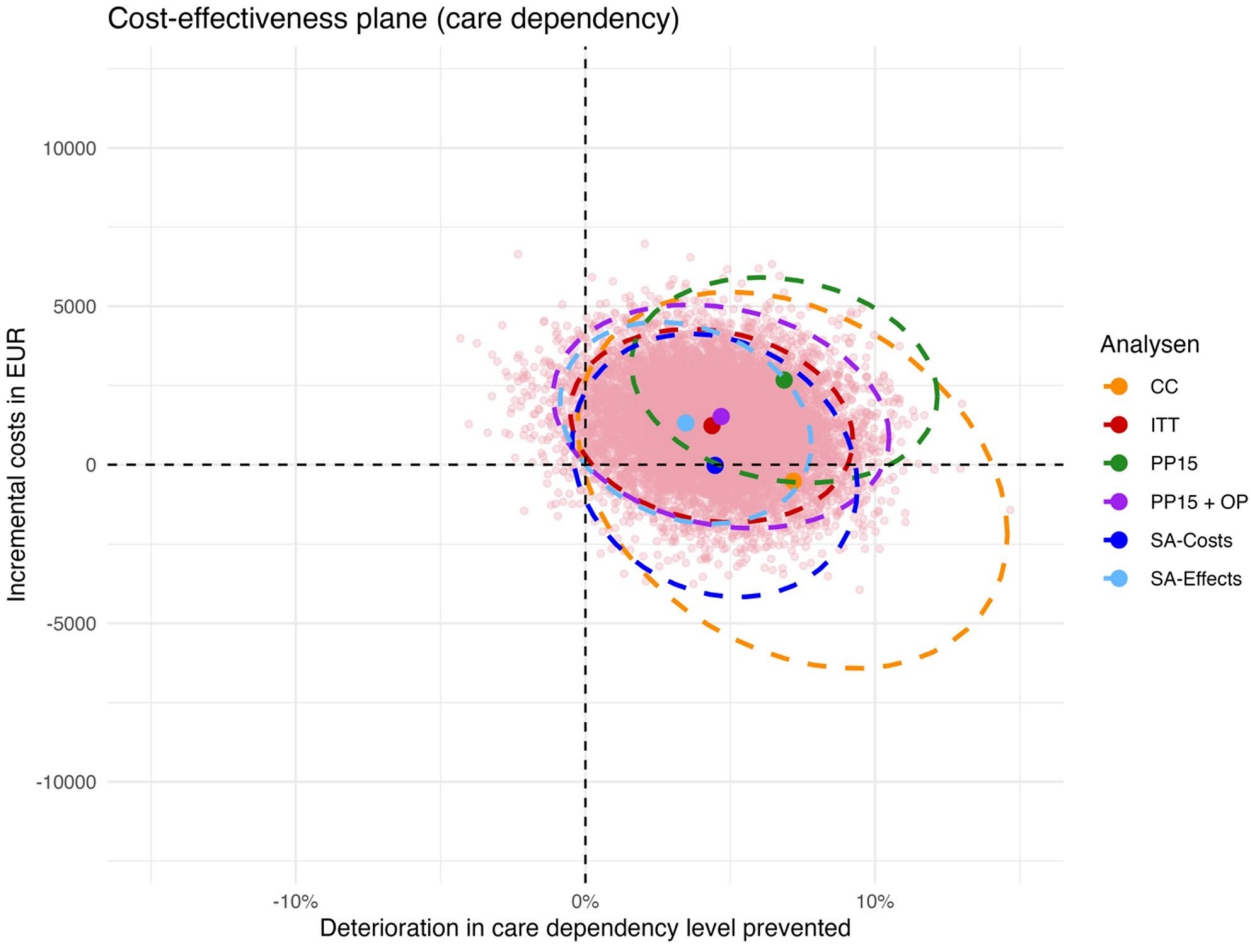
Cost-effectiveness plane showing a scatter plot of the ITT analysis and 95%-CI ellipses of all cost-effectiveness analyses based on the deterioration in care dependency level

Figure 2 shows a cost-effectiveness plane with a scatter plot of the ITT analysis in pink and the point estimate and 95%-CI ellipse in red as well as 95%-CI ellipses of all cost-effectiveness analyses based on the DCDL. As with the cost-utility analysis, most of the points are in the upper right quadrant with about one third of the points being in the lower right quadrant. The 95%-CI ellipse for the complete case analysis is also the largest here due to the smaller number of included patients. All ellipses crossed the horizontal axis for costs. The ITT, CC, PP15OP and SA ellipses also crossed the vertical axis for effects, whereas the PP15 ellipse did not, indicating that the intervention was significantly more effective in that non-randomised, exploratory per-protocol analysis.

### Cost-effectiveness based on WHODAS 2.0

The cost-effectiveness analysis based on the WHODAS 2.0 identified incremental costs of 1,203 EUR (bootstrapped 95% CI: -1,617; 3,976). The mean WHODAS 2.0 score at 12 months postoperatively was 26.06 ± 13.48 in the IG and 27.03 ± 14.48 in the CG, resulting in incremental effects of 0.97 points (bootstrapped 95% CI: -0.62; 2.54). This resulted in an ICER of 1,241 EUR per point gained. The sensitivity analysis excluding patients who had died resulted in mean costs of 26,560 EUR ± 25,116 in the IG and 25,260 EUR ± 24,258 in the CG, resulting in incremental costs of 1,300 EUR (bootstrapped 95% CI: -1,592, 4,169). The mean WHODAS 2.0 score at 12 months postoperatively was 23.51 ± 10.11 in the IG and 24.00 ± 10.95 in the CG, resulting in incremental effects of 0.49 points (bootstrapped 95% CI: -0.76; 1.73). This resulted in an ICER of 2,652 EUR per point gained.

### Cost-effectiveness acceptability

**Fig. 3:**
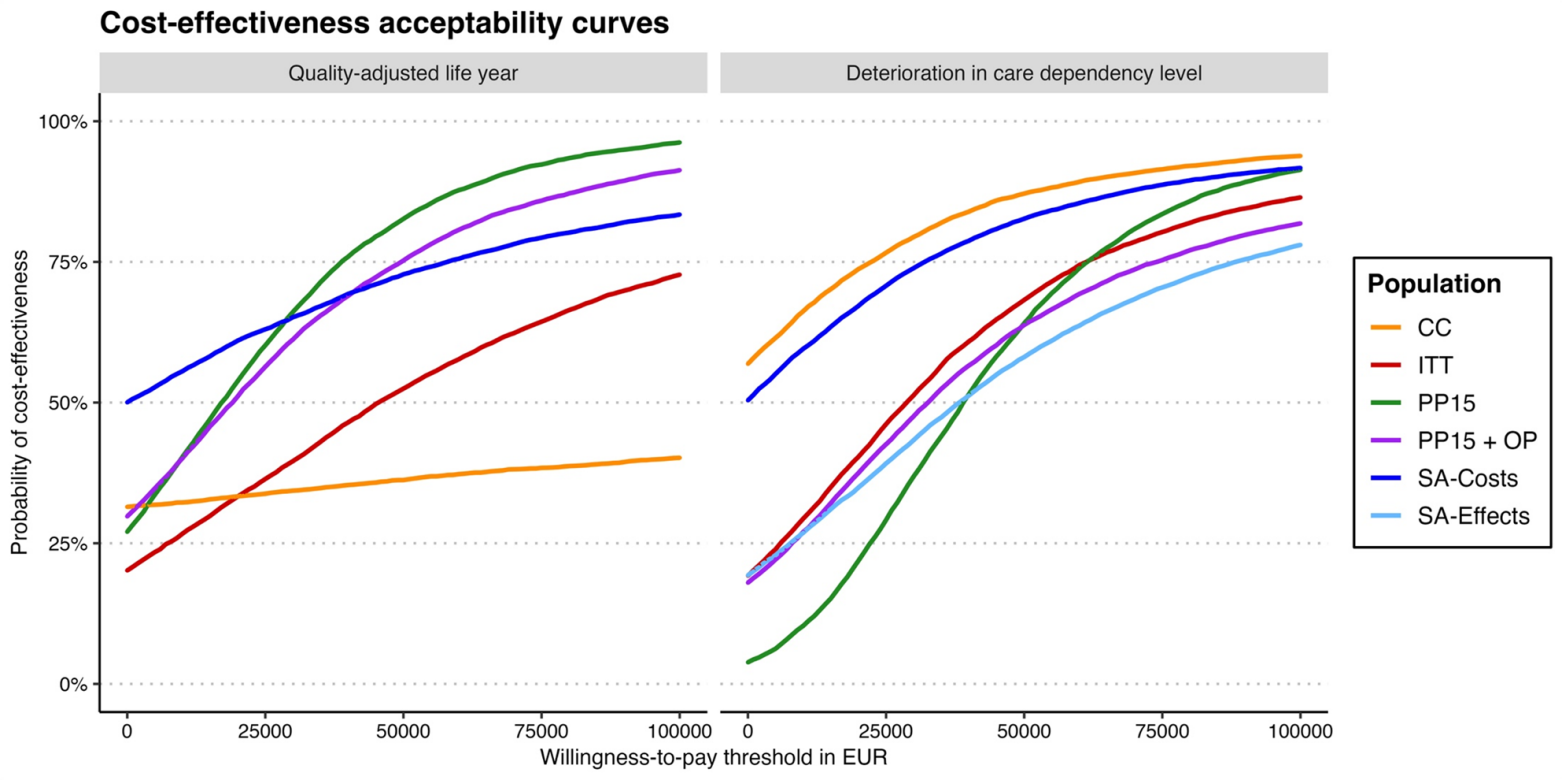
Overview of cost-effectiveness acceptability curves

Figure 3 shows the cost-effectiveness acceptability curves for the cost-utility analysis and the cost-effectiveness analysis based on the DCDL. At a willingness to pay of 0 EUR for an additional QALY, the probability that the intervention would be accepted as cost-effective was around 20% for the ITT. This increased to around 52% at a willingness to pay of 50,000 EUR, and to around 72% at 100,000 EUR. At a willingness to pay of 0 EUR for one patient with prevented DCDL, the probability that the intervention would be accepted as cost-effective was also around 20% for the ITT. This increased to around 69% at a willingness to pay of 50,000 EUR, and to around 86% at 100,000 EUR.

The highest cost-effectiveness acceptability at a willingness to pay of 0 EUR was observed for the sensitivity analysis based on non-winsorised cost data for the QALY (50%) and for the complete case analysis for the DCDL (57%). At a willingness to pay of 100,000 EUR, the highest cost-effectiveness acceptability was observed for the non-randomised, exploratory PP15 analysis for the QALY, with a probability of 96%, and for the complete case analysis for the DCDL, with a probability of 94%. In contrast, the complete case analysis for the QALY had by far the lowest cost-effectiveness acceptability at a willingness to pay of 100,000 EUR, with a probability of 40%.

### Subgroup analyses

The results of the subgroup analyses can be found in Additional file 1: Table S7 for the cost-utility analysis based on the QALY and in Additional file 1: Table S8 for the cost-effectiveness analysis based on DCDL. It should be noted that there were three subgroups with a small number of patients: Those who had undergone tumour surgery (*n* = 88), heart surgery or cardiac procedures (*n* = 111), and those who had undergone inpatient prehabilitation (*n* = 140 for the QALY analysis and *n* = 152 for the DCDL analysis).

The results for both outcomes were similar. The IG dominated the CG in three subgroups, where it was less costly and more effective. These subgroups were the pre-specified subgroup of patients who received prehabilitation in an outpatient setting (propensity-score matched, i.e. non-randomised, population), and the post hoc-defined subgroups of patients with a care dependency level of 0 at baseline and patients without cognitive impairment at baseline. Conversely, the CG dominated the IG in two subgroups, where the intervention was more costly and less effective: the subgroup of patients undergoing tumour surgery and the subgroup of patients receiving inpatient prehabilitation (propensity-score matched, i.e. non-randomised, population).

**Fig. 4:**
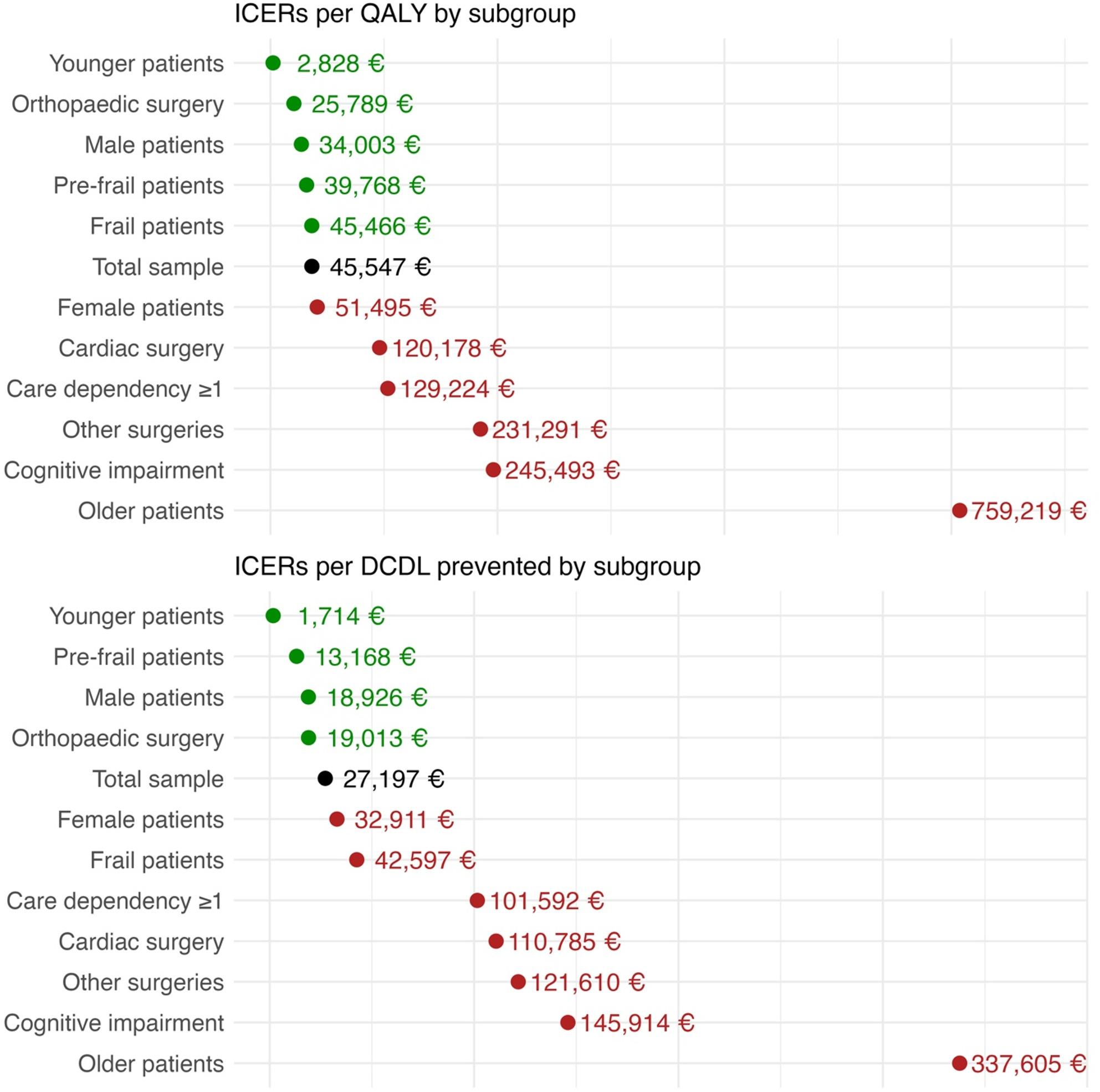
Incremental cost-effectiveness ratios of subgroups compared to the total sample

In patients undergoing cardiac surgery the intervention was also less effective, but also less costly. In the remaining subgroups, the intervention was more costly and more effective than the control, as in the main analysis. Compared to the ICERs for the total sample of 45,547 EUR per QALY and 27,197 EUR per patient with DCDL prevented, four subgroups had more favourable results (younger patients, male patients, patients undergoing orthopaedic or spine surgery and pre-frail patients) and six subgroups had less favourable results (older patients, patients with cognitive impairment at baseline, patients undergoing other types of surgeries, patient undergoing heart surgery or cardiac procedures, patients with a care dependency level at baseline of 1 or higher, and female patients) as shown in Fig. 4. Frail patients had a more favourable results than the total sample when looking at the QALY, but not for the DCDL. Examining the costs and effects separately revealed that the analyses by frailty status and care dependency level at baseline were primarily driven by the incremental costs of the intervention. In fact, the intervention was more effective for frail patients and those with a care dependency level of 1 or higher than for pre-frail patients and those with a care dependency level of 0.

## Discussion

### Summary of main results

This was an economic evaluation conducted alongside the PRAEP-GO trial [27], which compared an intervention consisting of frailty screening, a shared decision-making conference, and multimodal prehabilitation to standard preoperative care in a randomised cohort of 1,382 patients. We found that, from a societal perspective, prehabilitation tends to be more effective, but also more costly than standard care. This means that the probability of it being cost-effective largely depends on the willingness to pay.

The cost-utility analysis yielded an ICER of 45,547 EUR per QALY, indicating a 52% probability of cost-effectiveness at a willingness-to-pay threshold of 50,000 EUR. The cost-effectiveness analysis yielded an ICER of 27,197 EUR per patient with DCDL prevented, with a 69% probability of being cost-effective at 50,000 EUR. Due to the high uncertainty surrounding the results, a high probability (> 75%) was only observed at > 100,000 EUR and approximately 60,000 EUR, respectively. Regarding the QALY, the probabilities were higher in the per-protocol analyses, which included patients who received at least 15 units of prehabilitation (PP15) and those who additionally underwent the planned surgery (PP15OP). Furthermore, these probabilities increased more quickly and approached > 90% at 100,000 EUR. However, this was not reproducible for the DCDL, where the complete case analysis was most cost-effective at all willingness-to-pay thresholds. The cost-effectiveness analysis based on the WHODAS 2.0 yielded an ICER of 1,241 EUR per point gained. Whether this is considered cost-effective depends on the minimum clinically important difference, which has yet to be determined for a pre-frail or frail surgical population. Prespecified subgroup analyses revealed that the intervention is most cost-effective for patients who received prehabilitation in an outpatient setting. However, results of this analysis must be interpretated cautiously, as it was based on a propensity-score matched, i.e. non-randomised, population.

### Comparison with previous economic evaluations on prehabilitation

Our team has previously conducted a systematic review of the cost-effectiveness of prehabilitation [20], which identified 25 completed economic evaluations and 20 ongoing studies. Of the completed evaluations, six had a comparable analysis design to the present evaluation: four trial-based cost-utility analyses and two trial-based cost-effectiveness analyses. In two studies, cost-effectiveness was unclear as prehabilitation was more effective and more costly [65, 66]. One study did not report an ICER or a willingness-to-pay threshold [66]. In the other study, the converted ICER was 7,906 EUR (2020) per QALY gained, but no willingness-to-pay threshold was reported [65]. In the four remaining studies, prehabilitation was cost-effective based on the direction of the observed effects, as it was more effective and less costly [67–70]. Clinical effectiveness was slightly higher in these studies than in the present study, with incremental QALY or EQ-5D values ranging from 0.04 [68] to 0.12 [69]. It should be noted that all studies were conducted on an orthopaedic population, except for the study by Tew et al. [66], which included patients with abdominal aortic aneurysm repair. In the present economic evaluation, the incremental QALY of the orthopaedic subgroup (0.06, 95%-CI 0.02 to 0.10) was also higher than that of the total sample, which is consistent with the results of these evaluations [65, 67–70].

### Limitations

This economic evaluation was conducted in accordance with the highest methodological standards, including the pre-registration and publication of a detailed protocol in a peer-reviewed journal [28]. Nevertheless, some limitations must be considered when interpreting the results.

Firstly, as this was a piggy-back evaluation based on an RCT, the data used in the economic evaluation are subject to any bias that may have occurred in the trial, e.g., due to the lack of blinding participants to the intervention they received. Furthermore, the analyses may have been underpowered, as the sample size calculation was based on the PRAEP-GO trial’s primary outcome, and no post-hoc power analysis was performed. As the trial was conducted during the COVID-19 pandemic, it was affected by regional and national lockdowns and cancellations of planned surgeries, resulting in a complete halt of recruitment from October 30, 2020, to March 1, 2021 [27]. The organisation of prehabilitation with transportation was also affected by pandemic restrictions, as there were times when patients had to travel individually by taxi instead of being collected by shared transportation services, potentially leading to higher transportation costs. We did not account for the effects of pandemic restrictions in our analyses, however, as the situation was constantly changing and varied across regions, making it impractical to reliably quantify the effects.

Secondly, there is currently no scientifically accepted standard for the duration and frequency of prehabilitation. Thus, the prehabilitation programme used in the PREP-GO trial was based on the framework for early geriatric rehabilitation in Germany [71]. However, it is unclear whether the duration of three weeks and frequency of five supervised sessions per week was the ideal combination for maximum effectiveness of the programme, or whether a longer duration and lower frequency might have been more effective while still being feasible in the perioperative setting. Adherence in our sample was moderate, with 499 (81%) of 616 patients for whom prehabilitation was planned attending at least 15 sessions.

Thirdly, the costs of resource use during the 12-month postoperative follow-up period were measured using a patient-reported questionnaire tailored to elderly patients [48], which is susceptible to recall bias. Furthermore, a large proportion of patients in both groups were suspected of having a mild or severe neurocognitive disorder, which could have further impaired their recall. Consequently, the costs during follow-up may have been underestimated in both groups. In addition, there are no standardised unit costs for medications, and it was not possible to collect all the necessary information about the patients’ medications. For example, patients often did not know the pharmaceutical registration number, which prevented us from determining the cost of their medication. Nevertheless, medicines accounted for 17% of statutory health insurance expenditure in 2020, making them the third largest cost block after hospital stays and outpatient physician visits [72]. This suggests that the follow-up costs for both groups have been significantly underestimated, and that there may be significant differences in the mean total costs which are being obscured. We applied winsorisation to the costs of the index hospital stay and follow-up costs to reduce the influence of outliers. However, this altered the underlying data and thus the results; as shown by the sensitivity analysis of costs, the intervention group dominated the control group when the mean total costs were calculated without winsorisation. Overall, the cost estimates showed a high degree of uncertainty, as indicated by the wide confidence intervals around the mean difference estimates. However, as the multiply imputed datasets were combined prior to further analysis, true uncertainty may be even higher.

Fourthly, a large proportion of data was missing at the three-month follow-up visit, as this was not officially part of the trial, but was instead used predominantly to keep in touch with patients. For organisational reasons, the EQ-5D-5 L questionnaire was only administered if the visit was a house visit, not a telephone visit. Consequently, we had to impute a large number of values for this endpoint and visit, which may have distorted the combined QALY values from the four follow-up visits. Besides, the QALY was calculated based on the predicted utilities of the German general population, which may not adequately capture the preferences of elderly patients, as small improvements in function are not captured by the EQ-5D-5 L [73]. Nevertheless, a recent study found no systematic differences in published ICERs using QALYs when comparing individuals aged 65 years and older to those aged < 65 years [74].

Lastly, the care dependency level, which was chosen as the primary endpoint of the PRAEP-GO trial, is specific to the German healthcare system [44], which limits the international comparability of our findings. Furthermore, although the NBA is an established tool for determining care needs covered by insurance in Germany, the change in care dependency level comes with some methodological challenges and is not typically used as an endpoint in research studies. To simplify interpretation, we used the dichotomised version of deterioration versus no deterioration, which allowed us to calculate measures such as the difference in percentage difference (also known as absolute risk reduction). As there are no recognised thresholds for the minimal clinically important difference of disability measured using the DCDL or the WHODAS 2.0, the results of these analyses should be interpreted with caution, focusing on value for money rather than definitive cost-effectiveness.

### Generalisability and equity considerations

The PRAEP-GO trial applied broad inclusion criteria to achieve high generalisability of the results to the broader population of (pre-)frail surgical patients. However, the generalisability of the results may be somewhat limited by the fact that over half of the study participants had an indication for orthopaedic surgery. This was partly due to one of the participating hospitals specialising in orthopaedic surgery and enrolling 13.5% of the patients. In addition, the results cannot be readily transferred to other countries with different healthcare structures and reimbursement systems.

Furthermore, about 5% of patients had to be excluded after frailty screening because they met one or more exclusion criteria, although they, too, could have benefited from the intervention. While this decision was made for medical or practical reasons, it poses a threat to health equity. For example, patients with language barriers often belong to vulnerable groups, like migrants or refugees. Excluding these groups may reinforce existing disparities in access to healthcare, leading to poorer health outcomes. Therefore, when considering the implementation of prehabilitation into routine healthcare, it is critical re-evaluate whether the trial’s exclusion criteria should still apply. For example, there may be medical concerns regarding patients with severe heart or lung disease. In the case of patients with language barriers, the criterion should be lifted, and structural barriers, such as a lack of interpreters, should be addressed. This will ensure that prehabilitation programmes contribute to reducing, rather than inadvertently widening, health inequalities.

### Implications for future research, policy and practice

Future research on prehabilitation for (pre-)frail patients should focus particularly on evaluating the intervention in an outpatient setting, as exploratory subgroup analyses found it to be the most cost-effective setting in the present economic evaluation. This could involve considering digital support solutions for unsupervised sessions [75]. Furthermore, as cost-effectiveness was closely related to adherence to the prehabilitation programme regarding the QALY, any known barriers to participation should be addressed to optimise adherence [76]. Research on prehabilitation in general should include dose-response analyses to determine the optimal duration and frequency for a surgical population.

The present economic evaluation aimed to inform the potential reimbursement of the PRAEP-GO intervention within the statutory health insurance system in Germany. However, the probability of it being cost-effective largely depends on willingness to pay, and there is currently no binding willingness-to-pay threshold in Germany that policy makers must apply when deciding about the implementation of a new intervention. In the United Kingdom, for instance, the National Institute for Health and Care Excellence generally considers healthcare interventions to be cost-effective if they fall within a range of 20,000 to 30,000 GBP per QALY gained [77], which corresponds to approximately 22,300 to 33,400 EUR. The World Health Organization’s framework “Choosing Interventions that are Cost-Effective” (WHO-CHOICE) suggests that the country’s per capita gross domestic product (GDP) can be used as a threshold for very cost-effective healthcare interventions and a sum between 1- to 3-fold the GDP as cost-effective [78]. In 2020, Germany’s per capita GDP was approximately 41,500 EUR, resulting in probabilities of accepting the intervention as very cost-effective of 47% for the QALY and 63% for the DCDL and probabilities of accepting the intervention as cost-effective of over 72% and 86%, respectively. Overall, these figures would support the nationwide implementation of the intervention. Nevertheless, the WHO-CHOICE threshold values were primarily developed to categorise interventions into broad groups for consideration within a local context and are not meant to be used as a generic decision rule [79].

Finally, decisions regarding the implementation of the intervention also depend on their impact on the nation’s health budget, and the distributional considerations arising from spending a part of that budget on the intervention on a larger scale. A recent budget impact analysis from the Netherlands found that implementing a prehabilitation program for colorectal cancer surgery patients may lead to cost savings [80]. However, their model was based on the assumption that prehabilitation is both more effective and less costly, and therefore does not apply to the results of this economic evaluation. Future research should therefore include both budget impact analyses and distributional cost-effectiveness analyses to formally address health (in)equity impacts [81].

## Conclusions

The PRAEP-GO intervention was found to be more effective, but also more costly than standard preoperative care for elderly (pre-)frail patients prior to elective surgery. The probability of cost-effectiveness at an arbitrary willingness-to-pay threshold of 50,000 EUR was moderate (52% for the cost-utility analysis and 69% for the cost-effectiveness analysis). In the absence of a binding willingness-to-pay threshold in Germany, no clear recommendation can be made about future reimbursement of the intervention within the statutory health insurance system. Exploratory subgroup analyses showed that the intervention was cost-effective, i.e., more effective and less costly, for patients who received prehabilitation in an outpatient setting. Future research should therefore focus on delivering outpatient prehabilitation while implementation efforts should focus on optimising adherence by addressing barriers to participation as well as ensuring equitable access to maximise both clinical impact and societal value.

## Electronic Supplementary Material

Below is the link to the electronic supplementary material.

Supplementary material: Additional file 1: Tables S1-S9

## Data Availability

The datasets generated and/or analysed during the current study are not publicly available due to data protection regulations. The data and analysis code are available from the corresponding author on reasonable request, beginning one year after publication and continuing for up to five years.

## Abbreviations

%-diff: Difference in percentages
BMI: Body mass index
CC: Complete case analysis
CG: Control group
CI: Confidence interval
DCDL: Deterioration in care dependency level
ERAS: Enhanced recovery after surgery
EUR: Euro
GDP: Gross domestic product
IG: Intervention group
ICER: Incremental cost-effectiveness ratio
IQR: Interquartile range
ITT: Intention-to-treat
MD: Mean difference
MOCA: Montreal Cognitive Assessment
NBA: Neues Begutachtungsassessment (used to determine the care dependency level)
PP15: Per-protocol analysis of patients who had performed at least 15 supervised prehabilitation sessions
PP15OP: Per-protocol analysis of PP15 patients who additionally had undergone surgery
QALY: Quality-adjusted life year
SA: Sensitivity analysis
SD: Standard deviation
SMD: Standardised mean difference
WHO-CHOICE: World Health Organization’s framework Choosing Interventions that are Cost-Effective
WHODAS: World Health Organization Disability Assessment Schedule
