# Supplementary material for: Cost-effectiveness of prehabilitation for elderly (pre-)frail patients prior to elective surgery compared to standard care - an economic evaluation from a societal perspective
Source: BMC Med. 2026 May 21;24:326. doi: 10.1186/s12916-026-04933-6 (PMC13198052; doi:10.1186/s12916-026-04933-6)
Supplement: Supplementary file 1 — Supplementary material: Additional file 1: Tables S1-S9 [file 12916_2026_4933_MOESM1_ESM.pdf]

Supplementary material

Table S1: Changes from the published protocol

**Protocol section**	**Changes**
The PRAEP-GO RCT	None
Target population	None
Subgroups	We performed additional exploratory post-hoc analyses by level of care dependency at baseline and MOCA at baseline to determine the influence of these variables on the results.
Setting and location	None
Intervention and comparator	None
Perspective of the economic evaluation	We decided to publish the results from the payer and health care provider perspective separately as they used a different database (claims data from the statutory health insurance), resulting in significant differences in the methods employed.
Types of planned health economic evaluations	None (although the cost-effectiveness analysis from the payer perspective and health care provider perspective will be published separately (see above)).
Time horizon and discount rate	None
Health outcomes	Table 2: The data collection method of “Complications at discharge” (only relevant for provider perspective) was changed to medical documentation (patient charts, discharge letters) as this is more granular than routinely collected data. For valuation/ calculation only the Clavien-Dindo classification was used (a decision made on the trial level).
Resource use and costs	From the provider perspective, we had planned to calculate the difference between actual cost data and billing data. However, the actual cost data was not available on an individual level, so that we could not calculate this difference.<br>*Cost of intervention:* Instead of valuating the costs of outpatient prehabilitation based on based on duration, type of therapy and health care professional involved using the average national prices of 2022 for each billing code, we used the 2020 standardised unit costs by Muntendorf et al. 2024 [50] based on type of therapy to establish consistency with calculation of the follow-up costs. Costs of inpatient prehabilitation were based on diagnoses-related groups instead of outpatient billing codes plus overnight costs, as this more accurately reflects reality.<br>*Costs during follow-up:* Prices of pharmaceutical consumption could not be calculated due to incomplete documentation of patients’ use of medicines throughout the study.
Currency, price date, and conversion	The cost year was changed from 2022 to 2019/2020, since all unit costs, except those for patient transportation and the index hospital stay, originated from these years. No inflation or deflation adjustment was performed for transportation costs or the index hospital stay, so their price year was the billing year (ranging from 2020 to 2024). These costs were not adjusted because hospital costs in Germany are determined by cost weights for diagnosis-related groups, as well as a base rate which varies for each federal state and each year. As changes occur at both the weighting and base rate levels, there is no uniform percentage/rate for cost increases per year. Transportation costs were not adjusted due to their dependence on context-specific factors, including pandemic-related restrictions, which would have required complex, assumption-driven calculations.
Data management	None

Analytical methods	*Handling of missing data:* The BARMER data were not used for validation and completion of resource use and cost-data as they were only available for a subgroup of patients and aligning billing dates from administrative claims with the reference periods of the resource use questionnaire would have required extensive data linkage procedures and substantial additional analytical effort, with a high risk of misclassification.<br>*Primary and secondary analyses*: We did not perform secondary analyses with further imputation methods or an as-treated analysis, because only one patient from the control group was treated as part of the intervention group.<br>*Analysis plan - Sensitivity analyses:* We did not perform a model-based probabilistic sensitivity analysis, regression analyses for different distribution types of costs and effects, nor one-way sensitivity analyses for costs, as the results of non-parametric bootstrapping and a sensitivity analysis based on non-winsorised costs were considered sufficiently informative to characterise uncertainty. We did not assess the possible impact of the COVID-19 pandemic by comparing the results split into the periods of 2020/2021 and 2022/2023, as there had also been changes in the trial conduct and care settings that were unrelated to the pandemic, e.g. additional study centres and surgical departments as well as additional prehabilitation centres, making it impossible to isolate the impact of COVID-19.
Approach to engagement with different stakeholders	None
Trial status	None

Table S2: Measurement and valuation of resources and costs

Main block	Cost blocks	Resource unit	Resource valuation
Intervention costs	Frailty screening	Number of patients screened	Lump sum based on standard wages for physician assistants or nurses for 15 minutes of 19 EUR (2019, no conversion as study-specific values)
	Shared decision-making process	SDM preparation: mean duration in minutes	Lump sum based on standard wages for physicians of 66.60 EUR per hour (2019, no conversion as study-specific values) for mean duration
		SDM conference: number of professionals who participated in SDM conference (counting one per group) and if a family member participated	Lump sums based on standard wages by profession for 90 minutes (99.90 EUR for physicians, 54.00 EUR for a therapist or nurse; 2019, no conversion as study-specific values) and opportunity costs for family members (standardised unit costs*, substitution cost approach) for 90 mins of 45.39 EUR
	Outpatient prehabilitation	Number of sessions per modality<br>For mobile prehabilitation (at patient's home) also: Number of prehabilitation visits	Standardised unit costs* per modality and session: Physio- and sport therapy 21.45 EUR, occupational therapy 54.21 EUR, speech and language therapy 60.70 EUR, physician visit 73.13 EUR and psychotherapy 102.92 EUR. For nutrition counselling, study-specific costs (2019) were used: first session 90,57 EUR, next session(s) 67,94 EUR.<br>For mobile prehabilitation also: Travelling expenses for therapists per visit, lump sum per visit of 16.22 EUR (2019) based on benefit catalogue [83]
	Partial inpatient prehabilitation	Number of prehabilitation days	Standardised unit costs* for outpatient rehabilitation of 74.95 EUR per day
	Inpatient prehabilitation	Diagnosis-related group (DRG) and number of prehabilitation days	Lump sum per patient based on DRG cost weight for average geriatric early rehabilitative complex treatment (without diagnosis of acute stroke and additional procedure) of 5,356.52 EUR (2020), considering lower and upper limits for length of stay, plus cost weight for nursing care of 159.97 EUR per day
		Patient	Investment costs per patient of 331.68 EUR (2020)
	Patient transportation	Ride	Sum as invoiced as part of the study (price year = year of bill, range 2020 to 2024)
Index hospital stay	Hospital bill	Diagnosis-related group (DRG) and length of stay	Sum based on DRG, length of stay, intensive care unit (ICU) length of stay and extrabudgetary payments as invoiced by the hospital (price year = year of hospital stay, range 2020-2024) plus patient co-payment of 10 EUR (2020) per day for a maximum of 28 days
	Investment costs	Patient	Investment costs per patient of 331.68 EUR (2020)
Follow-up	Hospital (inpatient)	Days	Standardised unit costs* per day of 1,011.72 EUR on the ward and 2,192.49 EUR on the ICU
	Hospital (outpatient)	Days	Standardised unit costs* per day of 657.62 EUR

Main block	Cost blocks	Resource unit	Resource valuation
	Psychiatry	Days	Standardised unit costs* per day of 429.84 EUR
	Visits to physicians	Visit	Standardised unit costs* per specialty, ranging from 25.69 EUR for a general practitioner to 102.92 for a psychotherapist
	Visits to therapists	Visit	Standardised unit costs* per therapy group: Physiotherapy 21.45 EUR, occupational therapy 54.21 EUR, speech and language therapy 60.70 EUR, chiropody 37.98 EUR
	Mobile nursing care	Minute	Standardised unit costs* per minute of 0.59 EUR
	Informal care	Minute	Standardised unit costs* per minute using substitution cost approach of 30.26 EUR per hour
	Household assistance	Minute	Standardised unit costs* per minute of 0.42 EUR
	Help from neighbours etc.	Hours	Standardised unit costs* per minute using substitution cost approach of 30.26 EUR per hour
	Day care	Days	Standardised unit costs* per day depending on care dependency level, ranging from 33.79 EUR for level 1 to 90.02 EUR for level 5
	Short-term care	Days	Standardised unit costs* per day depending on care dependency level, ranging from 63.48 EUR for level 1 to 101.87 for level 5
	Nursing home	Days	Standardised unit costs* per day depending on care dependency level, ranging from 63.48 EUR for level 1 to 114.79 for level 5
	Rehabilitation	Days	Standardised unit costs* per day depending on setting, 74.95 EUR outpatient and 184.81 inpatient
	Auxiliary aids	Prescription	Standardised unit costs* per prescription, ranging from 60.58 EUR for walking aids to 3,285.76 EUR for tracheostomy aids

*Based on values for the year 2020 by Muntendorf et al. 2024 [50].

Table S3: Standardised mean differences for pre-specified baseline characteristics

Baseline characteristic	Standardised mean difference
Age	0.01
Sex	0.02
Charlson comorbidity index	-0.03
Index department	
Orthopaedics/trauma surgery	-0.03
General surgery	-0.03
Cardiology	0.03
Neurosurgery	0.01
Ophthalmology	0.02
ENT (ear, nose, throat)	0.01
Maxillofacial surgery	-0.01
Urology	0.00
Gynaecology	-0.02
Dermatology	0.00
Vascular surgery	0.00
Internal medicine	0.00
Cardiac surgery	0.01
Surgical risk	
High	0.00
Intermediate	-0.03
Low	0.03

Table S4: Costs following winsorisation, but without imputation

Cost block	Intervention group (n = 616)					Control group (n = 538)				
	Mean	SD	Median	Q1	Q3	Mean	SD	Median	Q1	Q3
**Intervention**	**2,414**	**2,837**	**1,546**	**1,025**	**2,122**	**Not applicable (0 EUR)**				
Frailty screening	26	0	26	26	26					
SDM conference*	309	116	332	287	387					
Prehabilitation*	2,079	2,808	1,195	644	1,749					
Mobile (n = 4)	831	155	798	747	882					
Outpatient (n = 69)	608	222	644	579	713					
Outpatient with transportation (n = 343)	1,360	577	1,277	1,006	1,580					
Part-inpatient (n = 23)	1,900	399	2,029	1,662	2,254					
Inpatient (n = 80)	9,069	924	9,056	8,883	9,217					
**Index hospital stay****	**11,218**	**8,448**	**8,203**	**6,553**	**11,882**	**11,800**	**9,040**	**8,265**	**6,474**	**13,069**
**12-month follow-up**	**5,088**	**13,445**	**121**	**0**	**2,780**	**4,956**	**13,277**	**61**	**0**	**1,934**
Hospital (inpatient)	5,141	10,350	0	0	5,185	5,042	10,030	0	0	6,070
Hospital (outpatient)	215	529	0	0	0	204	581	0	0	0
Psychiatry	0	0	0	0	0	0	0	0	0	0
Visits to physicians	599	436	532	301	803	618	496	533	273	837
Visits to therapists	632	672	409	59	976	574	681	343	0	850
Mobile nursing care	846	2,666	0	0	0	1,098	2,859	0	0	0
Informal care	3,198	11,859	0	0	0	3,807	14,031	0	0	0
Household assistance	127	386	0	0	0	174	458	0	0	0
Help from neighbours etc.	256	1,263	0	0	0	582	1,841	0	0	0
Day care	0	0	0	0	0	0	0	0	0	0
Short-term care	1	6	0	0	0	1	7	0	0	0
Nursing home	0	0	0	0	0	0	0	0	0	0
Rehabilitation	1,445	2,156	0	0	3,327	1,931	2,582	0	0	3,881
Auxiliary aids	224	432	61	0	165	237	477	0	0	121
**Total costs**	**19,908**	**20,063**	**12,671**	**9,021**	**23,756**	**17,730**	**20,287**	**10,649**	**7,401**	**20,951**

* This includes patients who did not receive an SDM conference or prehabilitation, for which the cost equalled 0 EUR.

** This includes patients who did not undergo surgery and thus had no index hospital stay, for which the cost equalled 0 EUR.

Abbreviations: SD, standard deviation; SDM, shared decision-making; Q1, first quartile; Q3 third quartile.

Table S5: Costs following imputation, but without winsorisation

Cost block	Intervention group (n = 616)					Control group (n = 538)				
	**Mean**	**SD**	**Median**	**Q1**	**Q3**	**Mean**	**SD**	**Median**	**Q1**	**Q3**
**Intervention**	**2,414**	**2,837**	**1,546**	**1,025**	**2,122**	**Not applicable (0 EUR)**				
Frailty screening	26	0	26	26	26					
SDM conference*	309	116	332	287	387					
Prehabilitation*	2,079	2,808	1,195	644	1,749					
Mobile (n = 4)	831	155	798	747	882					
Outpatient (n = 69)	608	222	644	579	713					
Outpatient with transportation (n = 343)	1,360	577	1,277	1,006	1,580					
Part-inpatient (n = 23)	1,900	399	2,029	1,662	2,254					
Inpatient (n = 80)	9,069	924	9,056	8,883	9,217					
**Index hospital stay****	**11,212**	**12,127**	**7,996**	**5,621**	**11,334**	**11,569**	**12,819**	**7,982**	**5,105**	**11,958**
**12-month follow-up**	**15,370**	**24,664**	**6,071**	**2,868**	**15,015**	**17,439**	**29,304**	**6,992**	**3,473**	**17,056**
Hospital (inpatient)	6,171	14,581	607	0	5,742	7,391	20,273	1,012	0	6,121
Hospital (outpatient)	172	590	0	0	0	181	791	0	0	0
Psychiatry	87	1,586	0	0	0	31	748	0	0	0
Visits to physicians	719	674	616	385	876	745	766	626	355	907
Visits to therapists	733	797	508	191	1,030	741	831	489	190	1,023
Mobile nursing care	1,509	8,515	0	0	418	1,713	8,541	0	0	475
Informal care	2,889	12,355	0	0	0	3,282	15,267	0	0	0
Household assistance	202	1,417	0	0	0	158	919	0	0	0
Help from neighbours etc.	507	2,676	0	0	0	706	3,433	0	0	0
Day care	16	156	0	0	0	45	440	0	0	0
Short-term care	51	520	0	0	0	62	745	0	0	0
Nursing home	429	2,560	0	0	0	233	1,764	0	0	0
Rehabilitation	1,647	2,201	684	0	3,248	1,893	2,658	1,072	0	3,040
Auxiliary aids	238	487	61	0	184	258	561	61	0	154
**Total costs**	**28,996**	**31,326**	**18,620**	**12,319**	**33,928**	**29,008**	**35,463**	**16,854**	**11,416**	**32,062**

* This includes patients who did not receive an SDM conference or prehabilitation, for which the cost equalled 0 EUR.

** This includes patients who did not undergo surgery and thus had no index hospital stay, for which the cost equalled 0 EUR.

Abbreviations: SD, standard deviation; SDM, shared decision-making; Q1, first quartile; Q3 third quartile.

Table S6: EQ-5D-5L utility values per follow-up visit following imputation

Timepoint	IG		CG	
	**Mean**	**SD**	**Mean**	**SD**
Baseline	0.63	0.29	0.63	0.30
3-month follow-up	0.70	0.30	0.69	0.30
6-month follow-up	0.74	0.30	0.70	0.32
9-month follow-up	0.73	0.29	0.71	0.31
12-month follow-up	0.71	0.33	0.68	0.36

Abbreviations: CG, control group; IG, intervention group; SD, standard deviation.

Table S7: Subgroup analysis of the quality-adjusted life year (QALY)

QALY	N		Costs IG		Costs CG		Incremental costs		Effects IG		Effects CG		Incremental effects		ICER
	IG	CG	Mean	SD	Mean	SD	MD	Bootstrapped 95% CI	Mean	SD	Mean	SD	MD	Bootstrapped 95% CI	EUR/QALY
Age < median	285	257	24,635	23,467	24,485	26,367	150	-4,074; 4,243	0.76	0.26	0.70	0.31	0.05	0.00; 0.10	2,828
Age ≥ median	331	326	28,330	26,320	26,153	23,160	2,178	-1,594; 6,125	0.69	0.29	0.69	0.28	0.00	-0.04; 0.05	759,219
Female	328	325	25,191	22,151	24,028	20,113	1,163	-2,025; 4,493	0.72	0.26	0.70	0.28	0.02	-0.02; 0.06	51,495
Male	288	258	28,249	28,012	27,168	29,272	1,080	-3,786; 5,870	0.71	0.30	0.68	0.31	0.03	-0.02; 0.08	34,003
Frail	228	214	31,076	31,429	28,738	23,339	2,338	-2,735; 7,552	0.60	0.31	0.55	0.32	0.05	0.01; 0.11	45,466
Pre-frail	388	369	24,002	20,056	23,492	25,160	510	-2,757; 3,632	0.79	0.23	0.78	0.24	0.01	-0.02; 0.05	39,768
Ortho surgery*	370	335	25,705	22,847	24,199	21,162	1,506	-1,743; 4,810	0.76	0.25	0.70	0.27	0.06	0.02; 0.10	25,789
Tumour surgery	53	35	31,139	40,627	19,227	20,885	11,912	-356; 25,458	0.63	0.34	0.80	0.23	-0.17	-0.29; -0.05	Control dominates
Heart surgery/ cardiac procedure	46	65	45,845	30,634	50,996	37,270	-5,150	-17,752; 7,498	0.55	0.37	0.59	0.35	-0.04	-0.18; 0.09	120,178
Other surgery	148	150	21,217	17,285	18,281	17,940	2,936	-1,212; 6,914	0.71	0.27	0.70	0.30	0.01	-0.05; 0.08	231,291
Outpatient setting**	422	422	23,738	21,876	25,207	25,718	-1,469	-4,736; 1,757	0.78	0.23	0.71	0.29	0.07	0.04; 0.11	Intervention dominates
Inpatient setting**	70	70	42,040	33,481	23,564	19,204	18,476	9,788; 28,069	0.57	0.32	0.69	0.28	-0.12	-0.21; 0.02	Control dominates
Care dependency = 0	469	422	22,722	19,829	22,850	22,428	-128	-3,019; 2,656	0.77	0.25	0.76	0.25	0.01	-0.03; 0.04	Intervention dominates
Care dependency ≥ 1	147	161	39,058	34,444	32,147	28,599	6,911	-143; 14,139	0.57	0.30	0.51	0.31	0.05	-0.02; 0.12	129,224
Cognitive impairment	409	387	28,956	27,518	26,673	23,509	2,283	-1,245; 5,894	0.67	0.30	0.66	0.31	0.01	-0.03; 0.05	245,493
Cognition normal	207	196	22,005	18,629	22,939	26,559	-934	-5,648; 3,417	0.82	0.19	0.76	0.24	0.06	0.02; 0.10	Intervention dominates

* Includes neurosurgical operations on the spine **Propensity score matched

Abbreviations: CG, control group; CI, confidence interval; IG, intervention group; ICER, incremental cost-effectiveness ratio; MD, mean difference; QALY, quality-adjusted life year; SD, standard deviation.

Table S8: Subgroup analysis of the deterioration in care dependency level (DCDL)

	N		Costs IG		Costs CG		Incremental costs		Effects IG		Effects CG		Incremental effects		ICER
**DCDL**	**IG**	**CG**	**Mean**	**SD**	**Mean**	**SD**	**MD**	**Bootstrapped 95% CI**	**N with DCDL**	**%**	**N with DCDL**	**%**	**%-diff**	**Bootstrapped 95% CI**	**EUR/ prevented DCDL**
Age < median	285	257	24,635	23,467	24,485	26,367	150	-4,074; 4,243	35	12.28	54	21.01	8.73	2.40; 15.00	1,714
Age ≥ median	331	326	28,330	26,320	26,153	23,160	2,178	-1,594; 6,125	73	22.05	74	22.70	0.65	-5.70; 7.15	337,605
Female	328	325	25,191	22,151	24,028	20,113	1,163	-2,025; 4,493	53	16.16	64	19.69	3.53	-2.40; 9.31	32,911
Male	288	258	28,249	28,012	27,168	29,272	1,080	-3,786; 5,870	55	19.10	64	24.81	5.71	-1.32; 12.74	18,926
Frail	228	214	31,076	31,429	28,738	23,339	2,338	-2,735; 7,552	61	26.75	69	32.24	5.49	-2.97; 14.01	42,597
Pre-frail	388	369	24,002	20,056	23,492	25,160	510	-2,757; 3,632	47	12.11	59	15.99	3.88	-1.15; 8.87	13,168
Ortho surgery*	370	335	25,705	22,847	24,199	21,162	1,506	-1,743; 4,810	48	12.97	70	20.90	7.92	2.40; 13.45	19,013
Tumour surgery	53	35	31,139	40,627	19,227	20,885	11,912	-356; 25,458	15	28.30	6	17.14	-11.16	-28.44; 6.86	Control dominates
Heart surgery/ cardiac procedure	46	65	45,845	30,634	50,996	37,270	-5,150	-17,752; 7,498	17	36.96	21	32.31	-4.65	-22.94; 13.33	110,785
Other surgery	148	150	21,217	17,285	18,281	17,940	2,936	-1,212; 6,914	28	18.92	32	21.33	2.41	-6.64; 11.69	121,610
Outpatient setting**	414	414	23,914	21,451	24,493	22,283	-579	-3,563; 2,454	52	12.56	87	21.01	8.45	3.36; 13.54	Intervention dominates
Inpatient setting**	76	76	46,033	36,478	24,179	22,387	21,854	12,558; 31,758	18	23.68	17	22.37	-1.32	-14.48; 11.92	Control dominates
CDL = 0	469	422	22,722	19,829	22,850	22,428	-128	-3,019; 2,656	76	16.20	82	19.43	3.23	-1.85; 8.19	Intervention dominates
CDL ≥ 1	147	161	39,058	34,444	32,147	28,599	6,911	-143; 14,139	32	21.77	46	28.57	6.80	-3.21; 16.64	101,592
Cognitive impairment	409	387	28,956	27,518	26,673	23,509	2,283	-1,245; 5,894	94	22.98	95	24.55	1.56	-4.38; 7.45	145,914
Cognition normal	207	196	22,005	18,629	22,939	26,559	-934	-5,648; 3,417	14	6.76	33	16.84	10.07	3.70; 16.28	Intervention dominates

* Includes neurosurgical operations on the spine **Propensity score matched

Abbreviations: CDL, care dependency level; CG, control group; CI, confidence interval; DCDL, deterioration in care dependency level; IG, intervention group; ICER, incremental cost-effectiveness ratio; MD, mean difference; SD, standard deviation.

Table S9: PRAEP-GO contributors

Type of contribution	Institute/Centre	Full names
PRAEP-GO consortium	**Charité – Universitätsmedizin Berlin** (executing entity: Department of Anesthesiology and Intensive Care Medicine (CVK/CCM))	Prof. Dr. med. Claudia Spies
	**BARMER Health Insurance** (executing entity: Institute for Health System Research)	Dr. med. Ursula Marschall
	**St. Joseph Krankenhaus Berlin-Tempelhof GmbH** (executing entity: Clinic for Geriatrics)	Dr. med. Rahel Eckardt-Felmberg
	**Hausarztpraxis Landgraf**	Dr. med. Irmgard Landgraf
	**Brandenburg Medical School Theodor Fontane** (executing entity: Institute of General Medicine)	Prof. Dr. med. Ulrich Schwantes
	**Technische Universität Berlin** (executing entity: Department of Healthcare Management)	Prof. Dr. med. Reinhard Busse
	**Ludwig-Maximilians-Universität Munich** (executing entity: Institute for Medical Information Processing, Biometry, and Epidemiology)	Prof. Dr. rer. nat. Ulrich Mansmann
Investigators	Charité - Universitätsmedizin Berlin, Berlin, Department of Anesthesiology and Intensive Care Medicine (CVK/CCM)	Daniela Adami, Dr. med. Olga Arbach, Birk Bähnemann, Dr. med. Stephan Bethe, Dr. med. Friedrich Borchers, Annemarie Büttner, Riccardo Di Rosso, Jeanette Duijst, Dr. med. Lukas Ehlen, Dr. med. Rebekka Epp, Lisa Eymold, Dr. med. Maria Fabian, Elke Falk, Barthel Georg, Tuba Gülmez, James Hilton, Laerson Hoff, Karoline Höfle, Elisa Iwan, Dr. med. Judith Kaden, Dr. med. Rainer Kuhly, Margret Külken, Dr. med. Oliver Kumpf, Dr. med. Florian Lammers-Lietz, Nadine Langer, Dr. med. Vincent Maertins, Dr. med. Rudolf Mörgeli, Dr. med. Anika Müller, Dr. med. Philipp Klassen-Beddig, Timo Kerlin, Dr. med. Anne Luise Reißhauer, Annett Römer, Karina Schirmeister, Dr. rer. nat. Kathrin Scholtz, Dr. med. Eva Schönenberger, Dr. med. Mareike Schulz, Dominik Schultze-Wolters, Dr. med. Maria-Anna Schütte, Natia Sichinava, Daniil Soflos, Olesia Ziesch, Dr. Björn Weiß
	Charité - Universitätsmedizin Berlin, Berlin, Department of Anesthesiology and Intensive Care Medicine (CBF)	Dr. med. Bernadette Kleikamp, Philipp Brandhorst, Dominik Steger, Dr. med. Golschan Asgarpur, Dr. med. Peter Fischer, Dr. med. Simon Sachse, Dr. med. Daniel Thierfelder
	A-K-tiv Therapiezentrum, Eutin	Anna-Lena H. Spiegel

	Anja Hense Physiotherapist, Kaltenkirchen	Anja Hense
	Arona Clinic for Geriatric Medicine, Berlin | Specialist Clinic for Geriatrics	Dr. med. Aylin Rohmann
	Ambulantes Rehazentrum Ottobrunn, Munich	Dr. med. Bernhard Papenfuß
	AMEOS Krankenhausgesellschaft Ostholstein mbH, Middelburg	Dr. med. Jens Dowideit
	Back to Activity München, Munich	Caroline Oefele
	BG Klinikum Unfallkrankenhaus Berlin, Berlin	Dr. med. Volker Gebhardt, Dr. med. Kristina Zappel
	Brandenburgklinik Berlin-Brandenburg, Bernau	Dr. med. Mehmet Gövercin
	Caritas Klinik Maria Heimsuchung Berlin, Berlin	Dr. med. Thomas König, Dr. med. Claudio Chesi
	Charité – Universitätsmedizin Berlin, Charité Physiotherapy and Prevention Centre, Berlin	Dr. med. Anett Reißhauer
	Charité – Universitätsmedizin Berlin, Clinic for Geriatrics and Geriatric Medicine	Adrian Rosada, Dr. med. Vavara Moskiou
	Evangelisches Geriatriezentrum Berlin, Berlin	Prof. Dr. med. Ursula Müller-Werdan
	Elithera, Neubrandenburg	Julia Brendicke
	Es.te Reha GmbH, Berlin	Brigitte Hartges
	Evangelisches Krankenhaus Hubertus Berlin, Berlin	Dr. med. Anja Heymann, Dr. med. Marion Hanke
	GLG ambulante Rehabilitation, Eberswalde	Dr. med. Slawomir Czuchra
	Herz- und Diabeteszentrum NRW, Bad Oeynhausen	Prof. Dr. med. Vera von Dossow
	Helios Amper-Kliniken, Dachau + Indersdorf	Dr. med. Björn Johnson
	KMG Elbtalklinik, Bad Wilsnack	Ines Dreger
	AMEOS-Klinik für Geriatrie Ratzeburg, Ratzeburg	Jens Leymann
	LMU University Hospital, Ludwig-Maximilians-Universität, Faculty of Medicine, Munich	Dr. Cordula Andres, Prof. Dr. Jörg Arnholdt, Ann-Cathrin Bischof, Dr. Maximiliane Burgmann, Marcus Gutmann, Dr. Anne-Marie Just, Prof. Dr. Eduard Kraft, Lina Lenninger, Dr. Giuseppe Magistro, Prof. Dr. Peter Müller, Tsitou Panagiota-Eirini, Dr. Alexander Paulus, Nora Petry, Prof. Dr. Simon Schäfer, Prof. Dr. Boris Schlenker, Dr. Vera Smolka, Dr. Elif Weidinger, Alina Winkler, Maike Zacharias
	medico concept GmbH, Rosenheim	Angelika Reisner
	Ostprignitz-Ruppiner Gesundheitsdienste GmbH, Neuruppin	Marco Liebsch
	Physiotherapy Meier, Munich	Franz-Josef Meier
	Physiotherapy, Fehmarn	Fabian Unger
	Physio Mölln, Mölln	Nicole Holst-Pitann, Erwin Oosterbaan
	Physio Power, Lübeck	Tobias Chyrek
	Physioline München, Munich	Wolf Leopold Albrecht
	Physiotherapy Anne Frische, Potsdam	Anne Frische

	Physiotherapy Köpenick, Berlin	Julia Wojciechowski
	Physiotherapy Lilli Günther, Carmzow-Wallmow	Lilli Günther
	Physiotherapy Schorrat, Lübbenau	Maik Schorrat
	Physiotherapy Wegener, Vetschau	Enrico Wegener
	Physiotherapy Wigger, Lübeck	Danny Wigger
	Praxis am Mühlentor, Templin	Marco Wollert
	Praxis Schmidke, Wittenberge	Tabea Schmidtke
	Rehaktiv, Aindling	Andy Cronauer
	Reha Day Clinic Berlin Pankow, Berlin	Martina Schuldt
	Giesinger + Schwabinger Reha-Sport GmbH&Co.KG, Munich	Michael Baum
	Reha-Zentrum Teltow GmbH&Co.KG, Teltow	Dr. med. Björn von Pickardt
	Sana Kliniken Sommerfeld, Kremmen	Dr. med. Agnieszka Deutschmann
	Sport- and Health Centre Rückenbalance GmbH, Königs Wusterhausen	Carsten Scholz
	Sport-REHA GmbH, Berlin	Lars May
	St. Joseph Krankenhaus Berlin, Berlin	Rahel Eckardt-Felmberg, Isabell Wenghöfer
	Sven Greyer- Physiotherapy and Fitness Studio, Storkow/Fürstenwalde	Sven Greyer
	Technical University of Munich, Munich	Alina Biegerl, Manfred Blobner, Kilian Klaus, Josef Blobner, Kay Eichelberg, Kristina Fuest, Sima Sattari
	therapiePUNKT, Munich	Michael Dummert
	University Medical Center Schleswig-Holstein, Lübeck	Carla Nau, Mareike Otto
	University Hospital, Greifswald	Anke Steinmetz
	University Hospital Eppendorf, Hamburg	Christian Zöllner, Cynthia Olotu
	University Hospital Ruppin Brandenburg, Ruppiner Kliniken GmbH, Neuruppin	Dr. med. Holger Stege
	VAMED Rehabilitationszentrum Lübeck, Lübeck	Ute Voß-Lümers
	Vivantes Auguste-Viktoria-Hospital, Berlin	Marc Kastrup
	Vivantes Hospital Kaulsdorf, Berlin	Dr. med. Katrin Knoll
	Vivental Rehabilitation Berlin, Berlin	Danny Lang
**Data Safety Monitoring Board**	Monash University, Melbourne, Australia	Carol Hodgson
	Charles University, Prague, Czech Republic	Frantisek Duska
	Shaare Zedek Medical, Center, Jerusalem, Israel	Sharon Einav
	Technical University of Munich, Munich, Germany	Kurt Ulm
